# Association of Food and Nonalcoholic Beverage Marketing With Children and Adolescents’ Eating Behaviors and Health: A Systematic Review and Meta-analysis

**DOI:** 10.1001/jamapediatrics.2022.1037

**Published:** 2022-05-02

**Authors:** Emma Boyland, Lauren McGale, Michelle Maden, Juliet Hounsome, Angela Boland, Kathryn Angus, Andrew Jones

**Affiliations:** 1 Department of Psychology, University of Liverpool, Liverpool, United Kingdom; 2 Department of Psychology, Edge Hill University, Ormskirk, United Kingdom; 3 Liverpool Reviews and Implementation Group, University of Liverpool, Liverpool, United Kingdom; 4 Institute for Social Marketing & Health, University of Stirling, Stirling, Scotland

## Abstract

**Importance:**

There is widespread interest in the effect of food marketing on children; however, the comprehensive global evidence reviews are now dated.

**Objective:**

To quantify the association of food and nonalcoholic beverage marketing with behavioral and health outcomes in children and adolescents to inform updated World Health Organization guidelines.

**Data Sources:**

Twenty-two databases were searched (including MEDLINE, CINAHL, Web of Science, Embase, and The Cochrane Library) with a publication date limit from January 2009 through March 2020.

**Study Selection:**

Preferred Reporting Items for Systematic Reviews and Meta-Analyses reporting guidelines were followed. Inclusion criteria were primary studies assessing the association of food marketing with specified outcomes in children and adolescents (aged 0-19 years). Exclusion criteria were qualitative studies or those on advertising of infant formula. Of 31 063 articles identified, 96 articles were eligible for inclusion in the systematic review, and 80 articles in the meta-analysis (19 372 participants).

**Data Extraction and Synthesis:**

Two reviewers independently extracted data. Random-effects models were used for meta-analyses; meta-regressions, sensitivity analyses, and *P* curve analyses were also performed. Where appropriate, pooling was conducted using combining *P* values and vote counting by direction of effect. Grading of Recommendations Assessment, Development, and Evaluation was used to judge certainty of evidence.

**Main Outcomes and Measures:**

Critical outcomes were intake, choice, preference, and purchasing. Important outcomes were purchase requests, dental caries, body weight, and diet-related noncommunicable diseases.

**Results:**

Participants totaled 19 372 from 80 included articles. Food marketing was associated with significant increases in intake (standardized mean difference [SMD], 0.25; 95% CI, 0.15-0.35; *P* < .001), choice (odds ratio, 1.77; 95% CI, 1.26-2.50; *P* < .001), and preference (SMD, 0.30; 95% CI, 0.12-0.49; *P* = .001). Substantial heterogeneity (all >76%) was unexplained by sensitivity or moderator analyses. The combination of *P* values for purchase requests was significant but no clear evidence was found for an association of marketing with purchasing. Data on dental health and body weight outcomes were scarce. The certainty of evidence was graded as very low to moderate for intake and choice, and very low for preference and purchasing.

**Conclusions and Relevance:**

In this systematic review and meta-analysis, food marketing was associated with increased intake, choice, preference, and purchase requests in children and adolescents. Implementation of policies to restrict children’s exposure is expected to benefit child health.

## Key Points

**Question:**

What is the association between food marketing (compared with less or no food marketing) and eating behavior and health in children and adolescents across the extant literature?

**Findings:**

In this systematic review and meta-analysis of 96 studies (64 randomized clinical trials, 32 nonrandomized studies), food marketing was associated with significant increases in food intake, choice, preference, and purchase requests. There was no clear evidence of associations with purchasing, and little evidence on dental health or body weight outcomes.

**Meaning:**

Results support the implementation of policies to restrict children’s exposure to food marketing.

## Teaser

This systematic review and meta-analysis quantifies the association of food and nonalcoholic beverage marketing with behavioral and health outcomes in children and adolescents.

## Introduction

Global trends show substantial increases in obesity among children in recent decades.^1^ This has serious implications for morbidity and mortality given that childhood obesity tracks into adulthood^2^ and excess weight is an important risk factor for noncommunicable disease (NCD).^3^ Changes in global systems are key drivers of rising obesity, specifically growth in the production of affordable, highly processed foods that are effectively marketed.^4^

Food and/or nonalcoholic beverage (hereafter referred to as *food*) marketing that largely promotes products high in fat, sugar, and/or salt (HFSS) is prevalent across television,^5^ digital media,^6^ outdoor spaces,^7^ and sport.^8^ Children and adolescents are particularly vulnerable to the effects of food marketing given their immature cognitive and emotional development, peer-group influence, and high exposure.^9,10^ The pathway linking exposure to HFSS food marketing with behavioral and health effects is complex^11^ but associations meet the criteria for a causal relationship.^12^ HFSS food marketing also negatively affects numerous child rights, including the right to the enjoyment of the highest attainable standard of health, the right to adequate food, and the right to privacy.^13^

Implementation of the World Health Organization (WHO) Set of Recommendations on the Marketing of Foods and Nonalcoholic Beverages to Children^14^ has been inconsistent.^13^ The underpinning evidence review^15^ largely predated the internet as a major marketing platform^16^ and there is more than a decade of new research to consider. Although its conclusions are corroborated by more recent reviews and meta-analyses,^17,18,19,20,21^ these are also limited to television advertising and dated digital marketing forms (eg, advergaming), including selective outcomes, such as intake, and lack assessment of evidential value or certainty. Therefore, WHO commissioned the current research to inform the development of updated recommendations to restrict food marketing to children.

## Methods

We conducted a systematic review and a series of meta-analyses following the Preferred Reporting Items for Systematic Reviews and Meta-analyses (PRISMA) reporting guideline.^22^ The WHO Nutrition Guidance Expert Advisory Group Subgroup on Policy Actions formulated the research question and identified the critical and important outcomes to be captured (eAppendix 1 in the Supplement). The terms *marketing*, *exposure*, and *power* were used as defined by WHO.^23^ The protocol was preregistered in the PROSPERO database in May 2019 (CRD42019137993).

### Search Strategy and Selection Criteria

We considered primary studies (randomized clinical trial [RCT] or nonrandomized study [NRS]) for inclusion if they assessed the association of food marketing with specified outcomes in children (aged 0-19 years). Exclusion criteria comprised qualitative designs and studies assessing the effect of advertising for infant formula or of marketing strategies outside of WHO’s definition. Critical outcomes comprised food intake, choice, preference, and purchasing (by, or on behalf of, children). Important outcomes were purchase requests (by children to a caregiver), dental caries and erosion, body weight, body mass index (BMI) and obesity, and diet-related NCDs (including validated surrogate indicators). Outcomes are defined in eAppendix 1 in the Supplement.

Searches were conducted in April 2019 and updated in March 2020 by an information specialist (M.M.). Data were analyzed in December 2020. Searches were limited to studies added to databases from January 1, 2009 (the previous global review included evidence to December 2008).^15^ We searched MEDLINE, CINAHL, Web of Science, Embase, ERIC, The Cochrane Library (CDSR, CENTRAL), Business Source Complete, EconLit, Emerald, JSTOR, HMIC, Advertising Education Forum, The Campbell Library, Database of Promoting Health Effectiveness Reviews (DoPHER), Healthevidence.org, TRIP, IRIS, Global Index Medicus, KOREAMED, Communication & Mass Media Complete, Academic Search Complete, and Index to Legal Periodicals & Books Full Text (H.W. Wilson). Targeted searches of Google and Google Scholar were undertaken. The search strategy is provided in the eAppendix 1 in the Supplement. All searches were peer reviewed (checked for accuracy by 3 researchers [E.B., L.M., K.A.] and a WHO librarian).

These searches were supplemented by (1) hand-searching reference lists of retrieved systematic reviews and eligible studies, (2) contact with topic experts, (3) forward and backward citation searching of included studies, and (4) a WHO evidence call for data.^24^ No language restrictions were applied.

Two reviewers (E.B., L.M., J.H., M.M.) independently screened studies against the inclusion criteria, assessing titles and abstracts to identify potentially relevant studies, then reviewing full texts. Titles and abstracts of articles not in English were screened using Google Translate, then researchers proficient in both languages translated the full texts for review. For multiple publications from the same cohort, we used data from the main contrast (food marketing vs no, less, or less powerful marketing) or the biggest sample. Disagreement was resolved through consensus and, if necessary, consulting a third reviewer. The search and screening processes were combined for this and a parallel review on the effectiveness of food marketing policies (Prospero identifier: CRD42019132506).

### Quality Assessment

We used Risk of Bias 2 to assess bias in RCTs and the Newcastle-Ottawa Scale to assess quality of the NRS. Bias assessments were conducted by one reviewer and independently checked by a second (E.B., L.M., J.H, M.M.).

### Statistical Analysis

Two reviewers (E.B., L.M.) independently extracted data using prepiloted forms. Study authors were contacted, if necessary, to provide data. Where data were only available in a figure, we used WebPlotDigitizer (version 4.3) for extraction.^25^

For studies with multiple interventions, we extracted data from all relevant interventions and the control group or most relevant comparator intervention. For studies with interventions comprising different levels of the same marketing exposures, we selected the largest (eg, most advertisements) as the exposure arm to maximize identification of effects. Relevant outcome measures and effect estimates were extracted. Where more than 1 eligible effect measure was available, we extracted the most comprehensive measure (eg, overall intake rather than of a single item) or prioritized the unhealthy categories.

Cochrane recommendations were followed for the synthesis.^26^ Meta-analysis was used where studies were sufficiently homogenous. Where meta-analysis was not possible, we selected the most appropriate synthesis method available: combining *P* values using Fisher method or vote counting by direction of effect (eAppendix 1 in the Supplement).

For meta-analyses, random-effects restricted maximum likelihood estimator analyses were conducted using the metafor package in R (version 4.1.3; R Foundation for Statistical Computing).^27^ The *I*^2^ (inconsistency) statistic was used to assess heterogeneity, with a value of *I*^2^ less than 50% indicating substantial heterogeneity. We undertook leave-one-out, trim and fill^28^ analyses, graphical displays of heterogeneity (GOSH), and Egger regression test to examine bias.^29^ We examined any influential cases with a difference in beta score more than 1.^30^

When appropriate, we conducted subgroup (moderation) analyses by study design (RCT vs NRS), marketing manipulation type (exposure vs power), and marketing channel (television vs digital vs packaging). Within RCTs, we examined whether risk of bias scores (low vs medium) moderated the association (not possible for the preference outcome owing to the small number of data points), and within NRSs we conducted meta-regressions to examine if scores on the NOS were associated with the effect. For diet and choice outcomes, we examined whether mean age of children in the sample or BMI *z* score of the sample was associated with effect size using meta-regression (not possible for the preference outcome owing to the small number of data points). To examine evidential value, we conducted *P* curve analyses using the dmetar function in R.^31^

We used Grading of Recommendations Assessment, Development, and Evaluation (GRADE)^32^ to judge the certainty of evidence as high, moderate, low, or very low (eAppendix 1 in the Supplement). Research team certainty assessments were revised where necessary following discussion with the WHO Nutrition Guidance Expert Advisory Group Subgroup on Policy Actions.

## Results

**Figure 1.**
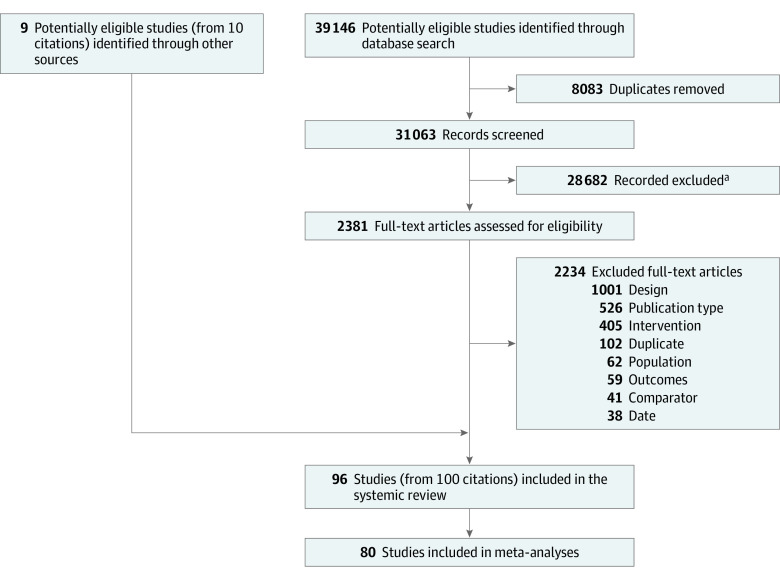
Study Selection PRISMA flow diagram detailing the study selection process. ^a^Reasons for exclusion: incorrect intervention, comparator, population, or date, duplicate records.

**Figure 2.**
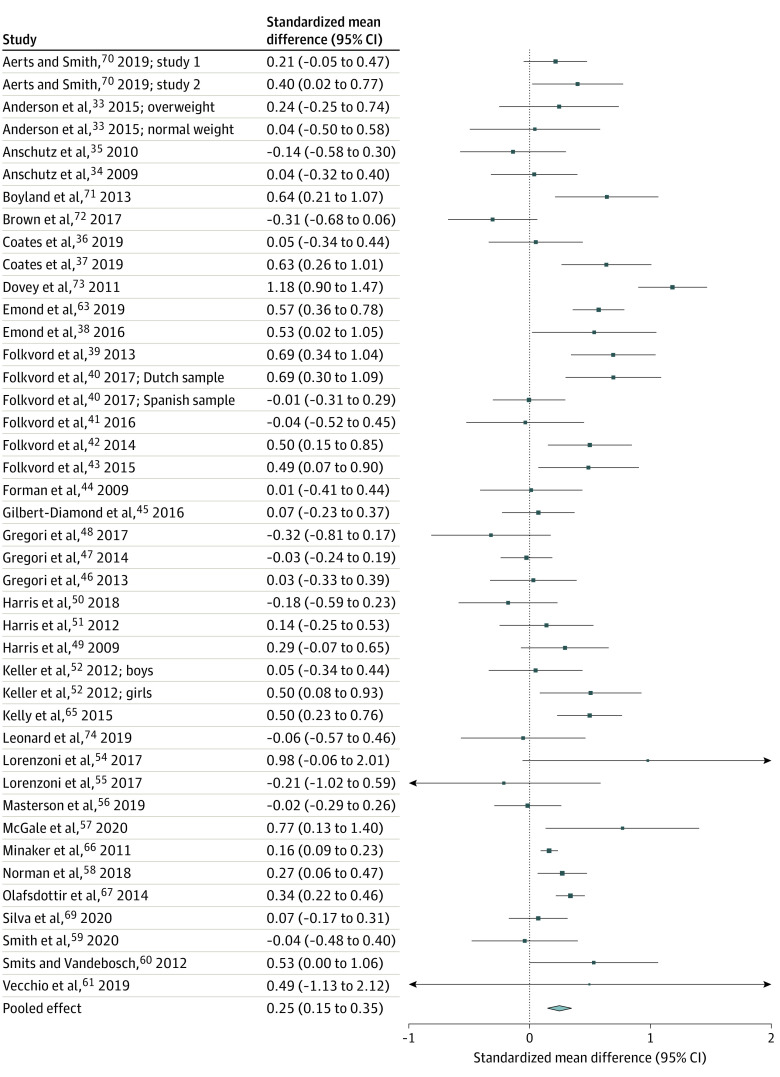
Forest Plot of Intake Data From Eligible Studies

**Figure 3.**
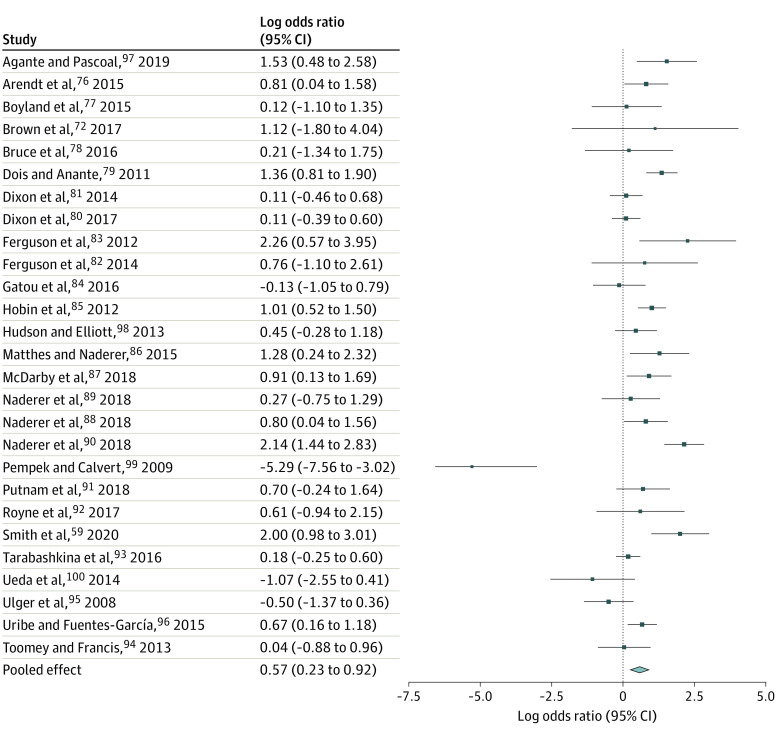
Forest Plot of Choice Data From Eligible Studies

**Figure 4.**
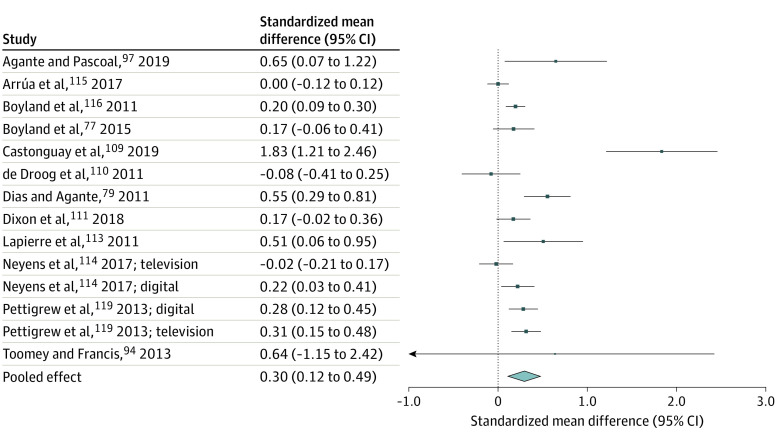
Forest Plot of Preference Data From Eligible Studies

A total of 31 063 titles were assessed for eligibility and 28 682 were ineligible (Figure 1). Of 2381 full-text articles assessed, 96 studies were included in the systematic review and 80 in the meta-analyses. Study characteristics are provided in the Supplement (eAppendix 2 in the Supplement). Pooled critical outcome data for food intake, choice, and preference are summarized in the Table. Overall forest plots are shown in Figure 2, Figure 3, and Figure 4. Forest plots for subgroup analyses, GOSH, and *P* curve plots are in eAppendix 4 in the Supplement.

**Table.** Pooled Associations of Food Marketing Compared With No Marketing, Less Food Marketing, or Less Powerful Food Marketing With Critical Outcomes

Study type	No. of studies	Participants, No./total No. (%)		Effect size (95% CI)	GRADE certainty of evidence
		Experimental arm	Control arm		
Nonrandomized studies					
Intake	11	4245/8436 (50.3)	4191/8436 (49.7)	SMD, 0.34 (0.12-0.57)^a^	Very low
Choice	5	261/416 (62.7)	155/416 (37.3)	OR, 0.56 0.05-5.99)^a^	Very low
Preference	4	1010/1972 (51.2)	962/1972 (48.8)	SMD, 0.21 (0.07-0.35)^b^	Very low
Randomized trials					
Intake	30	1456/2908 (50.1)	1452/2908 (49.9)	SMD, 0.20 (0.10-0.30)^a^	Moderate
Choice	22	1916/3838 (49.9)	1922/3838 (50.1)	OR, 1.97 1.46-2.66)^a^	Moderate
Preference	8	894/1802 (49.6)	908/1802 (50.4)	SMD, 0.38 (0.03-0.72)^b^^,^^c^	Very low

Abbreviations: GRADE, Grading of Recommendations Assessment, Development, and Evaluation; OR, odds ratio; SMD, standardized mean difference.

^a^ The high heterogeneity of the pooled effect size (≥50%) is unexplained by sensitivity analyses (although the association did not change direction or significance).

^b^ High heterogeneity (although the association did not change direction or significance).

^c^ Sensitivity analyses demonstrated that there was variability in the association when individual studies were removed but these analyses do not provide a public health–relevant explanation for the heterogeneity so downgrading for heterogeneity is still appropriate within the GRADE assessment.

Data relating to other outcomes, bias assessments, and all GRADE tables are in the Supplement (eAppendices 3, 5, and 6 in the Supplement). No relevant studies were identified with the diet-related NCDs outcome.

For food intake, 46 studies (in 43 articles) were identified (31 RCTs,^33,34,35,36,37,38,39,40,41,42,43,44,45,46,47,48,49,50,51,52,53,54,55,56,57,58,59,60,61^ 8 observational NRSs,^62,63,64,65,66,67,68,69^ and 7 experimental NRSs^70,71,72,73,74,75^). Pooled analyses of data from 41 studies (42 effect sizes) found that food marketing was associated with a significant increase in intake (standardized mean difference [SMD], 0.25; 95% CI, 0.15-0.35; *z* = 4.77; *I*^2^ = 77.2%; *P* < .001; Figure 2). The association was robust to sensitivity analyses and GOSH analyses demonstrated that across 100 000 iterations of the analyses the pooled effect SMD was approximately 0.24 (eAppendix 4 in the Supplement). There was no statistical evidence that study design (χ^2^ = 1.75; *P* = .19), marketing manipulation type (χ^2^ = 0.39; *P* = .53), or marketing channel (χ^2^ = 0.71; *P* = .70) significantly moderated the effect sizes. A meta-regression of mean age of children in the studies (mean [range] age, 8.6 years [4.1-13.6]) on the effect size was not significant (β = −0.02; 95% CI, −0.071 to 0.252; *P* = .35). There was no association between BMI *z* scores (mean [range],  1.01 [0.01-2.30]) and the effect size (β = 0.20; 95% CI, −0.136 to 0.534; *P* = .24). The *P* curve continuous test for evidential value was significant (*z* = 8.226; *P* < .001), indicating a true effect, as the distributions of *P* values were more frequent at *P* less than .01 compared with *P* of approximately .05. Of the 5 studies not included in the pooled analyses, 3 found associations of food marketing on intake^53,62,68^ and 2 found no association.^64,75^ The certainty of evidence for RCTs was moderate (affected by unexplained high heterogeneity), and for NRSs was very low (observational studies have a lower starting position within the GRADE assessment and certainty was downgraded owing to the imprecision of the effect size estimates from these studies).

For food choice, 37 studies (in 36 articles) were identified (27 RCTs^59,76,77,78,79,80,81,82,83,84,85,86,87,88,89,90,91,92,93,94,95,96^ and 10 experimental NRSs^72,97,98,99,100^). Pooled analyses of data from 27 studies found that food marketing was significantly associated with food choice (odds ratio [OR], 1.77; 95% CI, 1.26-2.50; *z* = 3.27; *I*^2^ = 77.5%; *P* < .001; Figure 3). Specifically, food marketing exposure was associated with increased odds of 1.77 times greater choice of the test item(s), irrespective of whether the test item was unhealthy or healthy. However, we note that only 3 of 27 effect sizes^85,87,99^ reported on choice of healthier items specifically and only 1 of those did so within a study design in which the marketing exposure itself was for healthier food.^85^ The association was robust to sensitivity analyses and GOSH analyses demonstrated that across 100 000 iterations of the analyses the pooled effect OR was approximately 1.70 (eAppendix 4 in the Supplement). There was no statistical evidence that study design (χ^2^ = 3.01; *P* = .08), marketing manipulation type (χ^2^ = 0.012; *P* = .91), or marketing channel (χ^2^ = 0.02, *P* = .99) significantly moderated the effect sizes. A meta-regression of mean age of children in the studies (mean [range] age, 8.76 [4.0–11.8] years) on the effect size was not significant (β = −0.08; 95% CI, −0.345 to 0.178; *P* = .53). The continuous test for evidential value was significant (*z* = 8.287; *P* < .001). Ten studies were not included in the pooled analysis; of these 8 found an association of food marketing with food choice (of which 7 were in the direction of greater choice of test items with food marketing exposure^74,101,102,103,104,105,106^ while 1 found greater choice of test items in the control condition^107^) and 2 found no association.^104,108^ Supplementary analysis of 3 of these studies^103,104^ that used a crossover design with binary outcomes showed a nonsignificant pooled OR of 3.45 (95% CI, 0.97-12.43). The certainty of evidence for RCTs was moderate (unexplained high heterogeneity), and for NRSs was very low (observational studies, risk of bias, and imprecision of the effect size estimates).

For food preference, 20 studies (in 19 articles) were identified (12 RCTs^53,77,79,94,104,109,110,111,112,113,114^ and 8 experimental NRSs^97,103,106,115,116,117,118,119^). Pooled analyses of data from 12 studies found that food marketing was significantly associated with increased food preference (SMD, 0.30; 95% CI, 0.12-0.49; Z = 3.21, *I^2^* = 90.0%; *P* = .001; Figure 4). The association was robust to sensitivity analyses and GOSH analyses demonstrated that across 100 000 iterations of the analyses the pooled effect SMD was approximately 0.53 (eAppendix 4 in the Supplement). There was no statistical evidence that study design (χ^2^[1] = 0.19; *P* = .67), marketing manipulation type (χ^2^ = 0.44; *P* = .51), or marketing channel (χ^2^ = 1.29; *P* = .53) significantly moderated the effect sizes. The continuous test for evidential value was significant (*z* = 5.504; *P* < .01). Eight studies were not able to be included in the pooled analysis, of which 6 found an association of food marketing with preference^2,53,103,104,106,112^ and 2 found no association.^117,118^ When studies with crossover designs and binary outcomes^103,104,106,112^ were analyzed separately, there was a significant association of marketing with preference (OR, 3.49; 95% CI, 2.03-6.22; *z* = 4.40; *P* < .001). The certainty of evidence for both RCTs and NRSs was very low (inconsistency, imprecision).

For food purchasing, 5 studies (1 RCT,^120^ 1 experimental NRSs,^121^ and 3 observational NRSs^66,122,123^) were identified. All 3 observational NRSs (moderate quality to high quality) found an association between food marketing and purchasing (2 effects of public health harm,^66,122^ 1 of public health benefit^123^). The RCT (with some concerns of bias)^120^ and moderate-quality experimental NRS^121^ found no association. The proportion of studies that found clear association of potential public health harm (1 of 4) was 25% (95% CI, 1.3%-78.1%). The proportion of studies that found unclear associations of potential public health harm (1 of 4) was 25% (95% CI, 1.3%-78.1%). The proportion of studies that showed any association (clear or unclear) of public health harm (2 of 5) was 40% (95% CI, 7.3%-83.0%). The certainty of evidence for both RCTs and NRSs was very low (risk of bias, inconsistency, imprecision).

For purchase requests, 6 studies (5 RCTs^60,81,110,111,114^ and 1 observational NRS^68^) were identified. The combination of *P* values was statistically significant in both model iterations (eAppendix 1 in the Supplement) suggesting evidence of food marketing associations with this outcome. The certainty of evidence for RCTs was moderate (risk of bias), and for NRS was very low (observational studies, risk of bias).

For dental caries, 2 observational NRSs were identified. A moderate-quality study found a clear association of public health harm^124^ and a high-quality study found no association.^69^ The proportion of studies that showed any association (clear or unclear) with public health harm (1 of 2) was 50% (95% CI, 9%-90.5%). The certainty of evidence was very low (risk of bias, inconsistency, indirectness).

Very little evidence was available on the association between food marketing and body weight or BMI. This review identified a single, moderate-quality observational NRS with no significant associations.^66^ The certainty of evidence was very low (risk of bias, indirectness). No studies were found with relevant data on diet-related NCDs or validated surrogate indicators.

## Discussion

In this study, food marketing exposure was associated with increases in children’s food intake, choice of and preference toward test items, and purchase requests. There was little evidence to support associations with food purchasing by or on behalf of children, while data relating to dental health and body weight outcomes were scarce. No studies were found for the diet-related NCDs or validated surrogate indicators outcome.

The effect sizes from the pooled analyses were small for intake and preference, moderate to large for choice, and robust to sensitivity analyses. *P* curve analyses demonstrated significant evidential value, indicative of a lack of selective reporting or *P* hacking. These findings are largely consistent with, and build on, previous findings,^15,17,18,20,21^ although there are some discrepancies. For example, Russell et al^20^ identified a moderating effect of BMI, such that children with overweight or obesity consumed an average of 45.6 kilocalories more than children with healthy weight following exposure to food advertisements. That type of subgroup analysis was not possible here owing to a lack of appropriate data reported in the studies (of the 5 effect sizes included in each group,^20^ 2 took place pre-2009, so were excluded here).

### Strengths and Limitations

A strength of the present review is that it has linked diverse formats of food marketing exposure (including newer digital forms, such as social media influencer marketing) to a range of behavioral and health outcomes. Other analyses have reported on a single format of marketing exposure (eg, screen-based) and fewer than 3 outcomes. The certainty of evidence for critical outcomes was most frequently rated as very low or moderate, which could be regarded as a limitation. However, as has been described previously,^125^ this reflects the nature of the GRADE criteria. GRADE prioritizes RCT data with clinical outcomes and requires certainty to be downgraded where there is unexplained heterogeneity, even where results are consistent between RCTs and NRSs and show similar findings to previous reviews, as here. The substantial observed heterogeneity, also consistent with previous meta-analyses,^17,18,20^ was unexplained by sensitivity analyses, or subgroup analyses on overall study design (although for intake and choice outcomes, variability was reduced when only RCTs were included), marketing manipulation, marketing format, study quality, participant age, or BMI. Therefore, this heterogeneity is likely a consequence of the large number of studies and more nuanced differences in study design (eg, stimulus types and outcome measurement). Substantial variability in outcome measurement is acceptable in meta-analysis but has implications for heterogeneity and therefore GRADE assessments.^126^

This study has limitations. As with the previous WHO review,^15^ much of the evidence lies at the proximal end of the spectrum (relative to a hierarchy of food marketing effects^11^) with data available on food intake, choice, and preference outcomes, but far less for the more distal outcomes (body weight and NCDs). Intake studies tend to measure immediate or short-term intake (directly following exposure to the marketing stimulus), rather than assessing diet across the day or longer term. Research gaps at the distal end likely reflect the substantial methodological challenge of conducting such studies, given that weight gain (or development of diet-related NCDs) typically occurs gradually and there is limited variability in the marketing exposure children experience within any given country or culture.^11^

The evidence is almost exclusively from higher-income countries, with only 6 studies conducted in lower-income to middle-income countries.^64,69,95,100,103,124^ The representativeness of the data for those populations may be limited and there was no opportunity to examine potential differences by income. Although we could explore the association of BMI and age with some outcomes through meta-regression, we could not conduct formal subgroup analyses by age (eg, child vs adolescent), socioeconomic status, gender, or rural/urban residential status owing to inadequate reporting (ie, insufficient studies with data segregated by these characteristics) and a lack of studies of adolescents. Future research should address this.

## Conclusions

This review provides a comprehensive update and quantitative synthesis of evidence of food marketing associations with critical behavioral outcomes and demonstrates the evidential value of these studies. WHO has previously recommended that member states enact policies to restrict children’s exposure to unhealthy food marketing^14^ and the review findings support this position.

## Notes

Supplement. eAppendix 1. Supporting information relating to the methodology eAppendix 2. Characteristics of included studies eAppendix 3. Risk of bias assessments eAppendix 4. Additional details on sensitivity and subgroup analyses relating to the food intake, choice, and preference outcomes eAppendix 5. Data for outcomes not suitable for meta-analysis and harvest plot for vote counting by direction of effect outcomes eAppendix 6: GRADE evidence profiles
