# Supplementary material for: Association of Food and Nonalcoholic Beverage Marketing With Children and Adolescents’ Eating Behaviors and Health: A Systematic Review and Meta-analysis
Source: JAMA Pediatr. 2022 May 2;176(7):e221037. doi: 10.1001/jamapediatrics.2022.1037 (PMC9062773; doi:10.1001/jamapediatrics.2022.1037)
Supplement: Supplement. — eAppendix 1. Supporting information relating to the methodology eAppendix 2. Characteristics of included studies eAppendix 3. Risk of bias assessments eAppendix 4. Additional details on sensitivity and subgroup analyses relating to the food intake, choice, and preference outcomes eAppendix 5. Data for outcomes not suitable for meta-analysis and harvest plot for vote counting by direction of effect outcomes eAppendix 6: GRADE evidence profiles [file jamapediatr-e221037-s001.pdf]

Supplemental Online Content

Boyland E, McGale L, Maden M, et al. Association of food and nonalcoholic beverage marketing with children and adolescents' eating behaviors and health: a systematic review and meta-analysis. *JAMA Pediatr.* Published online May 2, 2022.
doi:10.1001/jamapediatrics.2022.1037

eAppendix 1. Supporting information relating to the methodology

eAppendix 2. Characteristics of included studies

eAppendix 3. Risk of bias assessments

eAppendix 4. Additional details on sensitivity and subgroup analyses relating to the food intake, choice, and preference outcomes

eAppendix 5. Data for outcomes not suitable for meta-analysis and harvest plot for vote counting by direction of effect outcomes

eAppendix 6: GRADE evidence profiles

This supplemental material has been provided by the authors to give readers additional information about their work.

© 2022 Boyland et al. *JAMA Pediatrics*

Appendix 1: Supporting information relating to the methodology

PICO table

The PICO table was developed by NUGAG in Geneva in 2018.

Populations

Children (0-19 years). For food purchasing/sales studies only, studies of parents/other adults making food or non-alcoholic beverage purchases on behalf of children aged 0-19 years.

If possible, consider differences by equity characteristics (socioeconomic status, country of residence, age, gender etc.).

Interventions

Commercial food or non-alcoholic beverage marketing (not including food supplements, vitamins, or infant formula).

Comparison

Exposure to no, less, or less powerful (i.e., fewer techniques) marketing for foods and non-alcoholic beverages.

Outcomes

Outcome	Status of outcome	Definition of outcome
Food or non-alcoholic food intake	Critical	Food consumption, inclusive of lab-based objective measurement and reporting of habitual dietary patterns (e.g., frequency of consumption of fast food)
Food or non-alcoholic beverage choice or intended choice	Critical	Food category choice, brand choice, product choice, meal choice; measured using observation of choice behaviour e.g., child pointing, or hypothetical selection on paper or using computer-based tools.
Food or non-alcoholic beverage preference	Critical	The preferential selection, affected by numerous motives including liking, taste, perceived health benefits and price.
Food or non-alcoholic beverage purchasing/sales (by children or on behalf of children) or intended purchasing	Critical	Acquiring foods or non-alcoholic beverages through payment or expressing an intention to acquire such goods in the near future
Product requests or intended requests	Important	Children requesting, or expressing an intention to request, that parents/caregivers purchase a particular food or non-alcoholic beverage on their behalf
Dental caries/erosion	Important	Damage to teeth including loss of the surface of teeth
Body weight/body mass index/obesity	Important	The amount that a person weighs, or their weight status or calculated body mass index (kg/m^2).

© 2022 Boyland et al. *JAMA Pediatrics*

Diet-related non-communicable diseases (including validated surrogate indicators)	Important	Non-communicable diseases that have been related to unhealthy diets, including validated surrogate indicators such as HbA1c for diabetes.

© 2022 Boyland et al. *JAMA Pediatrics*

Example search strategy

Search terms were approved by a WHO librarian prior to finalisation. An example of a search strategy using keywords for MEDLINE is provided (adaptations were made as required for other databases):

Database: Ovid MEDLINE(R) ALL <1946 to April 25, 2019>

Search Strategy:

----

1 exp Food/ or exp Food Industry/ or beverages/ or carbonated beverages/ or energy drinks/ or "fruit and vegetable juices"/ or exp milk/ or exp milk substitutes/ or exp Tea/ or exp Teas, Herbal/ (1309168)

2 (food* or diet* or snack* or nutrition* or fast-food* or beverage* or drink*).ti. (403323)

3 Diet/ (150022)

4 1 or 2 or 3 (1572818)

5 Direct-to-Consumer Advertising/ or Advertising as Topic/ (14432)

6 exp Marketing/ (33888)

7 (advert* or advergame* or sponsor* or promot* or market* or adspend* or commercial or commercials).ti,ab. (1188923)

8 5 or 6 or 7 (1204370)

9 4 and 8 (104610)

10 exp adolescent/ or exp child/ or exp child, preschool/ or exp infant/ or exp schools/ (3471751)

11 (child* or adolescen* or infant* or youth* or "young people" or "young person" or teen* or pupil*).ti,ab. (1766353)

12 10 or 11 (3910602)

13 9 and 12 (13741)

14 ((food* or diet* or snack* or nutrition* or fast-food* or beverage* or drink*) adj3 (advert* or advergame* or sponsor* or promot* or market* or adspend* or commercial or commercials)).ab. (13252)

15 12 and 14 (3460)

16 13 or 15 (14527)

17 limit 16 to ed=20090101-20190426 (8271)

© 2022 Boyland et al. *JAMA Pediatrics*

Grading the certainty of evidence

The GRADE approach (Grading of Recommendations, Assessment, Development and Evaluation) was applied to assess the certainty of the available evidence for each outcome (GRADEpro software https://gradepro.org/).

GRADE focuses on the internal validity of bodies of evidence and is widely used in guideline development. Certainty of evidence can be graded as very low, low, moderate, or high. The evidence from observational studies start as being of low quality, while the evidence from randomised controlled trials starts as high quality. We considered five criteria for lowering the level of confidence: risk of bias, indirectness, imprecision, inconsistency, and likelihood of publication bias. Further, the level of confidence could be raised by three criteria: large effect, dose-response gradient, and where the influence of all plausible confounding would reduce a demonstrated effect or suggest a spurious effect when results show no effect (dose-response and plausible confounding were only considered where the evidence had not been downgraded on any domain for any reason), but this did not occur.

Where pooled analyses were not possible, for those outcomes we applied the constructs of GRADE in accordance with recommendations for rating the certainty of evidence in the absence of a single estimate of effect i.e., where data have been summarised narratively.[1] Where pooled analyses were undertaken for an outcome, we present the outcomes of the GRADE assessment in two formats: i) an assessment based on the single estimate of effect only and ii) this assessment and the non-pooled studies as a combined narrative summary. Results are presented in summary of findings tables, and we provide rationales for judgements in footnotes beneath the Evidence profile table.

© 2022 Boyland et al. *JAMA Pediatrics*

Combining p values and vote counting by direction of effect

Combining p values can be used where studies report no, or minimal information beyond p values and direction of effect. This approach answers the question ‘is there evidence that there is an effect in at least one study?’ but provides no information on the magnitude of effects.[2] For the product requests analysis, one effect had a significant p-value where the effect favoured the control. The analysis was conducted with and without this study included.

Because of a limited number of p values available, vote counting based on method of effect was employed for three outcomes in this review: purchasing/sales, body weight and dental outcomes. To conduct this synthesis, first we explored multiplicity within the data (where multiple effect measures of the same outcome domain were reported). As recommended by Cochrane for applying the vote counting based on the direction of effect synthesis method, we selected one effect measure per study per outcome to include in the synthesis. The selection was based on decision rules, namely (i) to first identify the most relevant effect measure in relation to the aims of the review, then (ii) if effect measures are equally relevant select the first reported effect. Only n=3 studies provided more than one effect measure for a single outcome, and in each case, both effects were equally relevant to the review aims so the first reported effect in each study was selected for synthesis. For the product requests analysis, one effect had a significant p value where the effect favoured the control. The analysis was conducted with and without this study included.

Five categories of effect direction were used in the review:

i. *Clear effect of public health harm*, where the effect estimate favours the intervention and the 95% CI excludes the null;
ii. *Unclear effect of potential public health harm*, where the effect estimate favours intervention but the 95% CI includes the null and is wide;
iii. *No difference in effect*, where the 95% CI crosses the null but is narrow;
iv. *Unclear effect of potential public health benefit*, where the effect estimate favours the control but the 95% CI includes the null and is wide; and
v. *Clear effect of public health benefit*, where the effect estimate favours the control and the 95% CI excludes the null.

If 95% CI were not reported, the p value was used to determine whether the direction of effect was clear or unclear (or if no difference existed, taken as p>.05) but never to determine the direction of effect. If no effect estimates or p value were reported, effects were always classified as ‘unclear’. Author reports of effect direction (e.g., if authors stated that one value was significantly greater than the other) and/or statistical significance (e.g., if no p value was reported but authors stated that no significant difference had been identified) were used to guide decisions.

We applied the binomial probability test on the (i) number of clear effects of public health harm and (ii) number of unclear effects of potential public health harm each compared to the number of effects clearly favouring the control, potentially favouring the control, or showing no effect. Analyses were conducted using the ‘prop.test’ function in R. The null hypothesis tested is that p=.5 (i.e., that there is an equal probability of effects of public health harm versus not). Therefore, the two-sided p value, based on Pearson’s chi-squared statistic, reflects the proportion difference between effects with undesirable effects (clear or potential public health harm) versus those with desirable effects (clear or potential public health benefit) or no effect. Significant p values can represent either a significantly *smaller* proportion of undesirable effects or a significantly *larger* proportion of undesirable effects compared with effects in the other categories. A non-significant p value is indicative of no significant differences in the proportions. Narrow CIs reflect more precise estimates of the proportion of interventions with desirable effects, due to an increased number of studies in the analysis. We also provide a combined harvest plot for the three outcomes synthesised in this way. The harvest plot is an effective, clear, and transparent way to portray evidence from a heterogeneous evidence base, especially where primary studies are not well-suited to statistical pooling.[3,4]

© 2022 Boyland et al. *JAMA Pediatrics*

Meta-analysis method

Meta-analyses were conducted for three outcomes: diet, food choice and food preference.

For the **diet and food preference** outcomes effect measures were continuous data so we computed the standardised mean difference [SMD = $mean^{exposure} - mean^{control}$ / pooled SD] and the standard error. The interpretation of the SMD was as follows: 0.2 was indicative of a small effect, 0.5 was indicative of a moderate effect, and 0.8 was indicative of a large effect[5]. In the current analysis, a positive SMD was indicative of greater consumption/preference after exposure to food marketing relative to a control condition. If the design was within subjects the standard error formula was adjusted by the correlation in line with Cochrane recommendations.[6] As the correlation between measurements was not readily available, we used r = .59, in line with previous research.[7] Where a standard error was reported in place of a standard deviation, we converted it using the formula SD = SE * √N.

Some studies had binary outcomes (e.g., child consumes cariogenic foods: yes or no) but otherwise fit our inclusion criteria. To include these studies, we computed the Odds Ratio and 95% confidence intervals and then converted the odds ratio to standardized mean difference using the formula SMD = logOR /1.81 from Chinn (2000)[8], and the variance of the SMD was calculated as variance of logOR*($3/3.1416^2$).

For the **diet** outcome, several studies provided quartile estimates rather than standard deviations, to convert these the formula Q3 – Q1/1.35 was used in line with Cochrane recommendations.[6]

For the **preference** outcome, where studies provided percentages in each group / condition, we calculated the raw numbers and if the percentages were not granular enough, we rounded the raw numbers to the nearest whole integer. If a study generated more than one effect size (e.g., two relevant experimental conditions, or two relevant effect measures for the same outcome) we took an average effect size. However, in the case of Pettigrew et al. (2013) we computed an effect size separately for TV and digital marketing exposure by dividing the N of the control group by 2 to allow for discrete subgroups.[9] Neyens et al. (2017) included both a TV and digital advertisement experimental condition (each compared to control). However, as the outcome was binary, we were unable to adjust the control group. The standard errors for this study will be narrower in this case, however removal of either of the effect sizes did not substantially influence the overall effect (as evidenced by leave-one-out analysis reported in the results section). One study (Toomey et al. 2013) had a 0 cell, therefore in line with recommendations[9] we added 0.5 to all cells in the 2 x2 table. Caution is advised as this often leads to overestimation of estimation of variance (as can be seen in the Forest Plot), which reduces the weight of the study. Several studies (n=5) included a cross-over design with a binary outcome (e.g., exposure to both experimental and control stimuli). In line with previous studies, these cross-over trials were poorly reported (e.g., condition totals only reported and not separated by A/B design (where intervention arm A is undertaken first by participants) and B/A design (where intervention arm B is undertaken first)).[10] Therefore, cross-over odds ratios were not able to be calculated.[11] To obtain an effect size we treated these studies as parallel trials to compute the log odds ratio and standard error. However, we did not include them in our main analyses (including subgroups), in line with Cochrane Recommendations, so results are reported separately.

For the **choice** outcome because effect measures provided binary choice data, log odds ratios were calculated and used in the meta-analysis. The standard error of the log odds ratios was calculated using the formula √(1/N[experimental group choice]+(1/N[experimental group no choice])+(1/N[control group choice])+(1/N[control group no choice]). If percentage choice rather than raw scores were reported we calculated the raw scores, if the percentage was not granular enough and did not lead to a whole integer we rounded as appropriate. Cross-over trials were not reported in enough detail (separately for A/B and B/A designs as described above), as such we were unable to calculate the appropriate precision.[11] Therefore, we treated these trials as parallel designs, but did not include them in the main analyses and instead ran a supplementary analysis on these studies (see results). Log odds ratios are converted using the exponential function to Odds Ratios for interpretation.

P curve analysis method

P curve examines the distribution of p values < .05 (based on Z-Scores).[12] If there was no effect of marketing on the outcome the distribution of p values would be uniform (no curve). If there is a true effect, the distributions of p values should be more frequent at p<.01 compared to p~.05 (right skew). If there is evidence of selective reporting (or p-hacking) then there will be a greater frequency of p values ~p=.05 (left skew). The continuous test is reported. This computes the p value for each significant p value from the individual effect sizes in the meta-analysis (the pp value). The pp values are standardized (Z-scored), the sum of the Z-Scores is divided by

© 2022 Boyland et al. *JAMA Pediatrics*

the number of tests and the resulting Z-score and corresponding value is the test for evidential value. We also report visual descriptions of the p curves.

Graphical Display of Heterogeneity (GOSH)[13]

GOSH performs separate meta-analysis on a number of subsets of the available data. In smaller meta-analyses this is usually all possible subsets, however here we chose to limit to 100,000 subsets. This means the meta-analysis was run 100,00 times using different iterations of included studies. From this we can examine the average effect size across the 100,00 models, the average I^2 statistic but also plot these to examine any influential studies.

Appendix 2: Characteristics of included studies

**Citation details**			**Study details**		**Outcomes reported**							**ROB**	**NOS**
**Lead author**	**Year**	**Country**	**Design**	**Marketing format**	**Diet**	**Choice**	**Pref.**	**Purch.**	**Requests**	**Body weight**	**Dental**		
Aerts	2019	Belgium	NRS	Packaging	✓								S1 = 8<br>S2 = 5
Agante	2019	Portugal	NRS	Digital		✓	✓						3
Anderson	2015	Canada	RCT	TV	✓							Some concerns (both studies)	
Anschutz	2009	Netherlands	RCT	TV	✓							Some concerns	
Anschutz	2010	Netherlands	RCT	TV	✓							Some concerns	
Arendt	2015	Austria	RCT	TV		✓						Some concerns	
Ares	2016	Uruguay	NRS	Packaging		✓							4
Arrua	2017	Uruguay	NRS	Packaging			✓						6
Boyland	2011	UK	NRS	TV			✓						8
Boyland	2013	UK	NRS	TV	✓								10
Boyland	2015	UK	RCT	TV		✓	✓					Some concerns	
Brown	2017	US	NRS	TV	✓	✓							12
Bruce	2016	US	RCT	TV		✓						Some concerns	
Castetbon	2012	US	NRS	TV				✓					8
Castonguay	2019a	US	RCT	TV			✓					High risk	
Coates	2019a	UK	RCT	Digital	✓							Low risk	
Coates	2019b	UK	RCT	Digital	✓							Some concerns	
Dalton	2017	US	NRS	TV	✓								7
De Droog	2011a	Netherlands	RCT	Packaging			✓		✓			High risk	
Dias	2011	Portugal	RCT	Digital		✓	✓					Some concerns	
Dixon	2014	Australia	RCT	Packaging		✓			✓			Low risk	
Dixon	2017	Australia	RCT	Packaging		✓						Some concerns	
Dixon	2018	Australia	RCT	Packaging			✓		✓			Some concerns	
Dovey	2011	UK	NRS	TV	✓								8
Drewnowski	2017	France	NRS	Other				✓					5
Emond	2016	US	RCT	TV	✓							Some concerns	
Emond	2019b	US	NRS	TV	✓								7
Ferguson	2012	US	RCT	TV		✓						Some concerns	
Ferguson	2014	US	RCT	TV		✓						Some concerns	
Fernandez	2019	Malaysia	NRS	TV, Digital	✓								4

© 2022 Boyland et al. *JAMA Pediatrics*

Folkvord	2013	Netherlands	RCT	Digital	✓							Some concerns	
Folkvord	2014	Netherlands	RCT	Digital	✓							Some concerns	
Folkvord	2015	Netherlands	RCT	Digital	✓							Some concerns	
Folkvord	2016a	Netherlands	RCT	Digital	✓							Some concerns	
Folkvord	2017	Netherlands	RCT	Digital	✓							Some concerns (both studies)	
Forman	2009	US	RCT	Packaging	✓							Some concerns	
Gatou	2016	Greece	RCT	TV		✓						Some concerns	
Ghimire	2013	India	NRS	TV							✓		4
Gilbert-Diamond	2017	US	RCT	TV	✓							Some concerns	
Gregori	2013	Italy	RCT	TV	✓							Low risk	
Gregori	2014	Italy	RCT	TV	✓							Some concerns	
Gregori	2017a	Italy	RCT	TV	✓							Some concerns	
Gunnarsdottir	2010	Iceland	RCT	Packaging			✓					Some concerns	
Harris	2009	US	RCT	TV	✓							Some concerns	
Harris	2012	US	RCT	Digital	✓							Some concerns	
Harris	2018	US	RCT	TV	✓							Some concerns	
Hartmann	2017	Germany	NRS	Packaging			✓						7
Heard	2016	US	RCT	Packaging				✓				Some concerns	
Hobin	2012	Canada	RCT	Packaging		✓						Some concerns	
Hudson	2013	US	NRS	TV		✓							4
Jones	2011	Australia	RCT	Print		✓						Some concerns	
Keller	2012	US	RCT	Packaging	✓							Some concerns	
Kelly	2015	Australia	NRS	TV	✓								5
Kotler	2012	US	RCT	Packaging	✓		✓					Some concerns	
Lapierre	2011	US	RCT	Packaging			✓					Low risk	
Leonard	2019	US	NRS	Packaging	✓	✓							5
Letona	2014	Guatemala	NRS	Packaging		✓	✓						8
Levin	2010	US	NRS	Packaging			✓						3
Lorenzoni	2017a	Italy	RCT	TV	✓							Some concerns	
Lorenzoni	2017b	Italy	RCT	TV	✓							Some concerns	
Masserot	2010	France	NRS	TV	✓								6
Masterson	2019	US	RCT	TV	✓							Some concerns	
Matthes	2015	Austria	RCT	TV		✓						Some concerns	
McAlister	2012	US	NRS	Packaging		✓							5
McDarby	2018	Ireland	RCT	Packaging		✓						Some concerns	

© 2022 Boyland et al. *JAMA Pediatrics*

McGale	2016	UK	RCT	Packaging		✓	✓					Some concerns (both studies)	
McGale	2020	UK	RCT	Packaging	✓							Some concerns	
Minaker	2011	Canada	NRS	Packaging	✓			✓		✓			5
Naderer	2018a	Germany	RCT	TV		✓						Some concerns	
Naderer	2018b	Germany	RCT	TV		✓						High risk	
Naderer	2018c	Germany	RCT	TV		✓						Some concerns	
Neyens	2017	Belgium	RCT	TV, Digital			✓		✓			Some concerns	
Norman	2018a	Australia	RCT	TV, Digital	✓							Low risk	
Ogle	2017	US	RCT	Packaging		✓						Some concerns	
Olafsdottir	2014	Sweden	NRS	TV	✓								5
Pempek	2009	US	NRS	Digital		✓							4
Pettigrew	2013	Australia	NRS	TV, Digital			✓						3
Putnam	2018	US	RCT	Digital		✓						Some concerns	
Reimann	2017	US	RCT	Packaging		✓						High risk	
Roberto	2010	US	NRS	Packaging		✓	✓						7
Royne	2017	US	RCT	TV		✓						Some concerns	
Scully	2012	Australia	NRS	TV, Digital, Outdoor	✓				✓				5
Silva	2020	Brazil	NRS	TV	✓						✓		8
Simões	2014	Portugal	NRS	Print				✓					5
Smith	2020	Australia	RCT	Digital	✓	✓						Some concerns	
Smits	2012	Belgium	RCT	Packaging	✓				✓			Some concerns	
Tarabashkina	2016a	Australia	RCT	Digital		✓						Low risk	
Toomey	2013	US	RCT	TV		✓	✓					Some concerns	
Ueda	2014	Brazil	NRS	TV		✓							8
Ülger	2009	Turkey	RCT	TV		✓						Some concerns	
Uribe	2015	Chile	RCT	TV		✓						Some concerns	
Vecchio	2019	Italy	RCT	TV	✓							Some concerns	

© 2022 Boyland et al. *JAMA Pediatrics*

Appendix 3: Risk of bias assessments

RCTs

Lead author	Year	Randomization process	Deviations from intended interventions	Missing outcome data	Measurement of the outcome	Selection of the reported result	Overall
Anderson	2015	?	?	+	+	+	!
Anschutz	2009	?	+	+	+	+	!
Anschutz	2010	?	?	+	+	+	!
Arendt	2015	?	+	+	+	+	!
Boyland	2015	?	?	+	+	+	!
Bruce	2016	?	+	+	+	+	!
Castonguay	2019a	?	−	+	+	?	−
Coates	2019a	+	+	+	+	+	+
Coates	2019b	?	?	+	+	?	!
De Droog	2011a	?	−	?	+	?	−
Dias	2011	?	?	+	+	?	!
Dixon	2014	+	+	+	+	+	+
Dixon	2017	?	+	+	+	+	!
Dixon	2018	?	+	+	+	+	!
Emond	2016	?	?	+	+	+	!
Ferguson	2012	?	+	+	+	?	!
Ferguson	2014	?	+	+	+	?	!
Folkvord	2013	?	+	+	+	+	!
Folkvord	2014	?	+	+	+	+	!
Folkvord	2015	?	+	+	+	+	!
Folkvord	2016a	?	+	+	+	+	!
Folkvord	2017	?	+	+	+	+	!
Forman	2009	?	+	+	+	+	!
Gatou	2016	?	+	+	+	+	!
Gilbert-Diamond	2017	?	+	+	+	+	!
Gregori	2013	+	+	+	+	+	+
Gregori	2014	?	?	+	+	+	!
Gregori	2017a	?	+	+	+	+	!
Gunnarsdottir	2010	?	+	+	+	?	!
Harris	2009	?	+	+	+	?	!
Harris	2012	?	+	+	+	+	!

Key

+ Low risk
! Some concerns
- High risk

© 2022 Boyland et al. *JAMA Pediatrics*

Lead author	Year	Randomization process	Deviations from intended interventions	Missing outcome data	Measurement of the outcome	Selection of the reported result	Overall
Harris	2018	?	?	+	+	+	!
Heard	2016	?	?	+	+	+	!
Hobin	2012	?	+	+	+	+	!
Jones	2011	?	?	+	+	?	!
Keller	2012	?	?	+	+	?	!
Kotler	2012	?	+	?	+	?	!
Lapierre	2011	+	+	+	+	+	+
Lorenzoni	2017a	?	+	+	+	+	!
Lorenzoni	2017b	?	?	+	+	+	!
Masterson	2019	?	+	+	+	+	!
Matthes	2015	?	+	+	+	+	!
McDarby	2018	?	+	+	+	+	!
McGale	2016	?	+	+	+	+	!
McGale	2020	?	+	+	+	+	!
Naderer	2018a	?	+	+	+	+	!
Naderer	2018b	–	?	+	+	?	–
Naderer	2018c	?	?	+	+	+	!
Neyens	2017	?	+	+	+	?	!
Norman	2018a	+	+	+	+	+	+
Ogle	2017	?	+	+	+	?	!
Putnam	2018	?	?	+	+	?	!
Reimann	2017	–	+	+	+	?	–
Royne	2017	?	+	+	+	+	!
Smith	2020	?	?	+	+	+	!
Smits	2012	?	+	+	+	+	!
Tarabashkina	2016a	+	+	+	+	+	+
Toomey	2013	?	?	+	+	!	!
Ülger	2009	?	?	+	+	+	!
Uribe	2015	?	?	+	+	+	!
Vecchio	2019	?	?	+	+	+	!

Key

+ Low risk
! Some concerns
- High risk

© 2022 Boyland et al. *JAMA Pediatrics*

Cohort studies

	Selection				Comparability		Outcome			
**Study name**	**Representative**	**Selection of non-exposed cohort**	**Ascertainment of exposure**	**Outcome not present**	**Comparability a**	**Comparability b**	**Assessment of outcome**	**Length of follow-up**	**Adequacy of follow-up**	**Total score**
Boyland 2011	b	a	b	No	age, bmi		a	a	a	8
Boyland 2013	b	a	a	Yes	Interaction of age, weight, BMI		a	a	a	10
Brown 2017	b	a	a	Yes	Adjusted for age, BMI	Also adjusted for income, race, and baseline hunger	a	a	a	12
Hartmann 2017	b	a	a	Yes	NR	NR	a	a	a	7
Pettigrew 2013	d	a	c	No	NR	NR	c	a	a	3
Simões 2014	b	a	c	Yes	NR	NR	c	a	a	5

NR = not reported

N/A = not applicable

a, b, c, d = these letters denote the study quality rating for each item across all domains on the NOS scale, with ‘a’ indicating the highest quality.

© 2022 Boyland et al. *JAMA Pediatrics*

Cross sectional studies

	Selection				Comparability	Outcome		
**Study name**	**Representativeness of the sample**	**Sample size**	**Non-respondents**	**Ascertainment of the exposure (risk factor)**	**Comparability**	**Assessment of outcome**	**Statistical test**	**Total score**
Aerts 2019 (1)	b	b	c	a	a	b	a	8
Aerts 2019 (2)	d	b	c	a	b	b	a	5
Agante 2019	c	b	c	c	b	b	a	3
Ares 2016	c	b	c	a	b	c	a	4
Arrua 2017	c	b	a	b	b	b	a	6
Castetbon 2012	a	b	c	a	a	a	a	8
Dalton 2017	b	b	b	b	a	b	a	7
Dovey 2011	a	b	c	a	a	b	a	8
Drewnowski 2017	a	c	a	a	b	c	a	5
Emond 2019	b	b	b	b	a	b	a	7
Fernandez 2019	b	b	c	c	b	b	a	4
Ghimire 2013	c	b	c	b	b	b	a	4
Hudson 2013	c	b	c	b	b	b	a	4
Kelly 2016	b	b	c	c	a	c	a	5
Leonard 2019	b	b	c	b	b	b	a	5
Letona 2014	b	b	b	a	a	b	a	8
Levin 2010	b	b	c	a	N/A	d	b	3
Masserot 2010	d	b	c	b	a	b	a	6
McAlister 2012	b	b	a	b	b	b	a	5
Minaker 2011	b	c	c	c	a	c	a	5
Olafsdottir 2014	a	b	b	c	a	c	a	5
Pempek 2009	b	b	c	b	b	b	b	4

© 2022 Boyland et al. *JAMA Pediatrics*

	Selection				Comparability	Outcome		
Study name	Representativeness of the sample	Sample size	Non-respondents	Ascertainment of the exposure (risk factor)	Comparability	Assessment of outcome	Statistical test	Total score
Roberto 2010	a	c	b	a	a	c	a	7
Scully 2012	a	b	b	c	a	c	a	5
Silva 2020	b	a	b	b	a	a	a	8
Ueda 2014	b	a	c	b	a	b	a	8

NR = not reported

N/A = not applicable

a, b, c, d = these letters denote the study quality rating for each item across all domains on the NOS scale, with ‘a’ indicating the highest quality.

© 2022 Boyland et al. *JAMA Pediatrics*

Appendix 4: Additional details on sensitivity and subgroup analyses relating to the food intake, choice, and preference outcomes

Food Intake

Sensitivity analyses

Leave-one-out analyses demonstrated little variability in the effect when individual studies were removed (SMDs ranged 0.22-0.26), all models were statistically significant (ps<.001). Trim and fill analyses did not identify any hypothetical studies to impute, and no studies had a DFBETA value >1. Egger's regression test was also not significant (Z=0.13, p=.89).

GOSH analysis

The average I^2 across the 100,000 models was 70.85% and the average effect size was SMD=.246. All pooled effect sizes were positive, as can be seen from the histogram (eFigure 1).

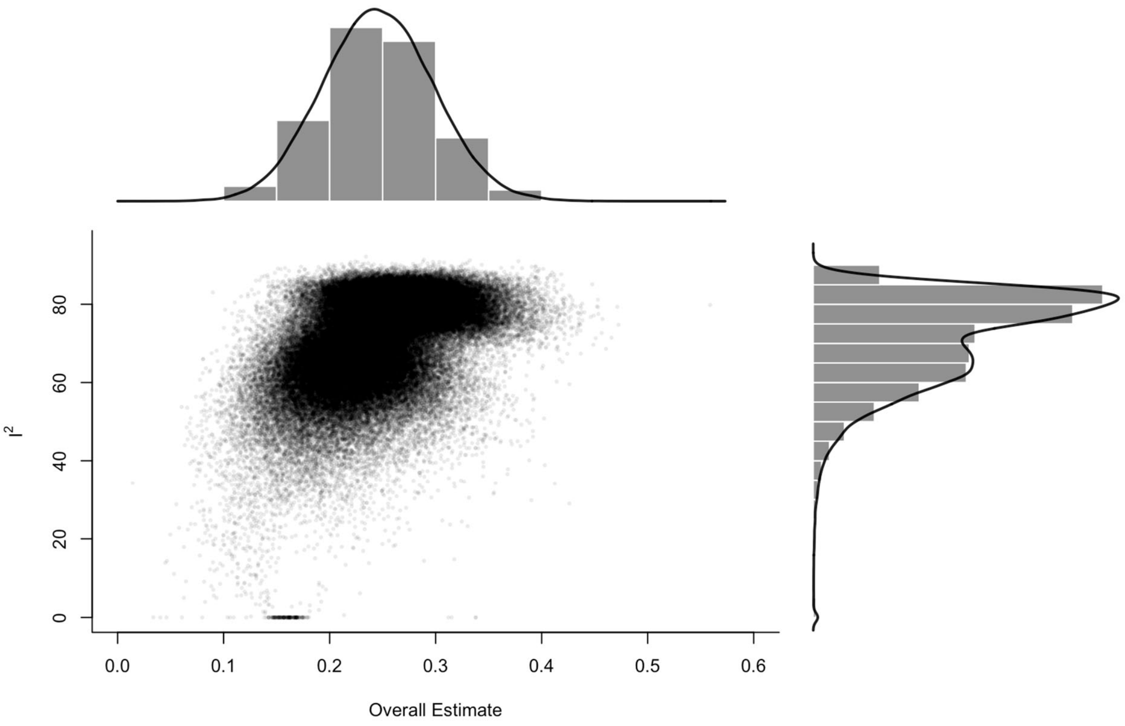

eFigure 1: GOSH plot for food intake meta-analysis

Subgroup analyses by risk of bias

There was no statistical evidence that study bias significantly moderated effect sizes in RCTs ($X^2(1)=0.19$, p=.66) and there was no statistical evidence of an association between bias scores and effect sizes in NRS (B=-.02, p=.69).

Subgroup analyses by study design

The effect size for RCTs (N = 31 eFigure 2) was SMD=0.20 [95% CI: .09 to .30: I^2=51.5%], the effect size for NRS (N=11; eFigure 3) was SMD=0.34 [95% CI: .12 to .57: I^2 = 92.2%].

© 2022 Boyland et al. *JAMA Pediatrics*

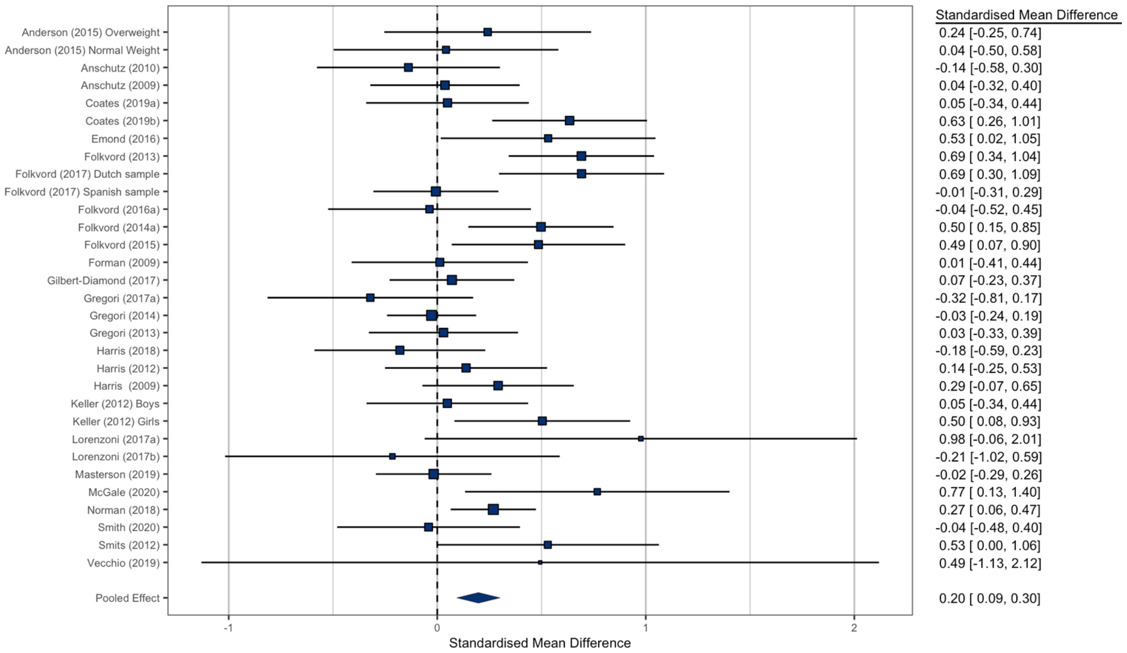

eFigure 2: Forest plot for pooled analyses of RCTs with food intake outcomes

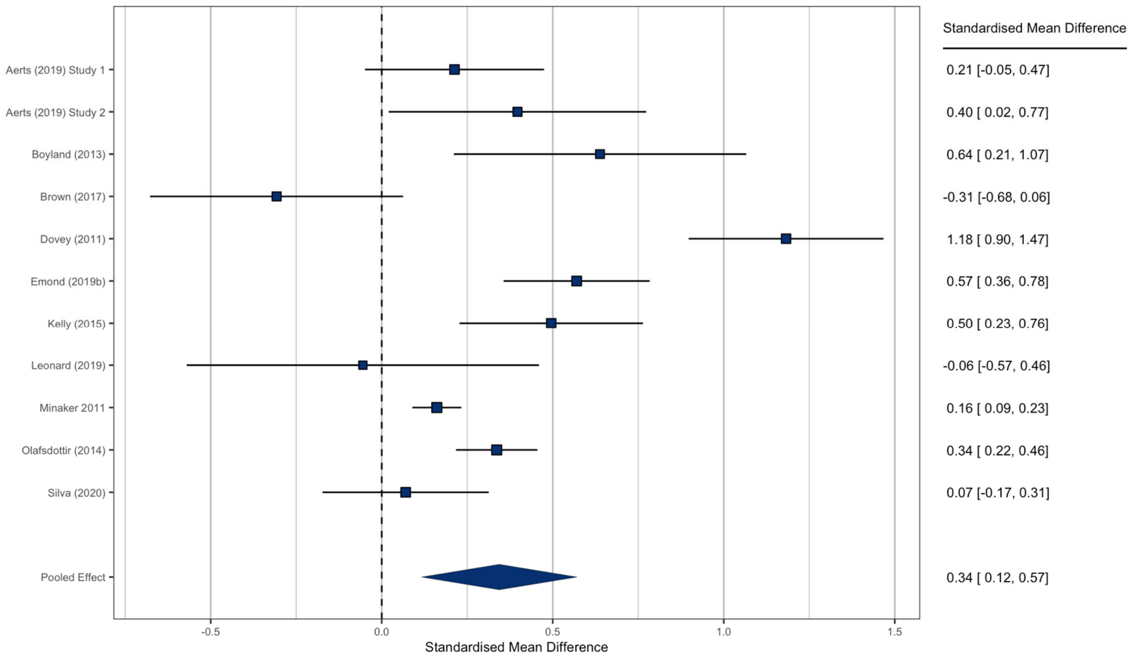

eFigure 3: Forest plot for pooled analyses of NRS with food intake outcomes

© 2022 Boyland et al. *JAMA Pediatrics*

Subgroup analyses by marketing manipulation type

The effect size for exposure was SMD=0.24 [95% CI: .12 to .35: I^2=79.7%], the effect size for power was SMD = 0.30 [95% CI: .12 to .49: I^2=4.1%]. See eFigures 4 and 5.

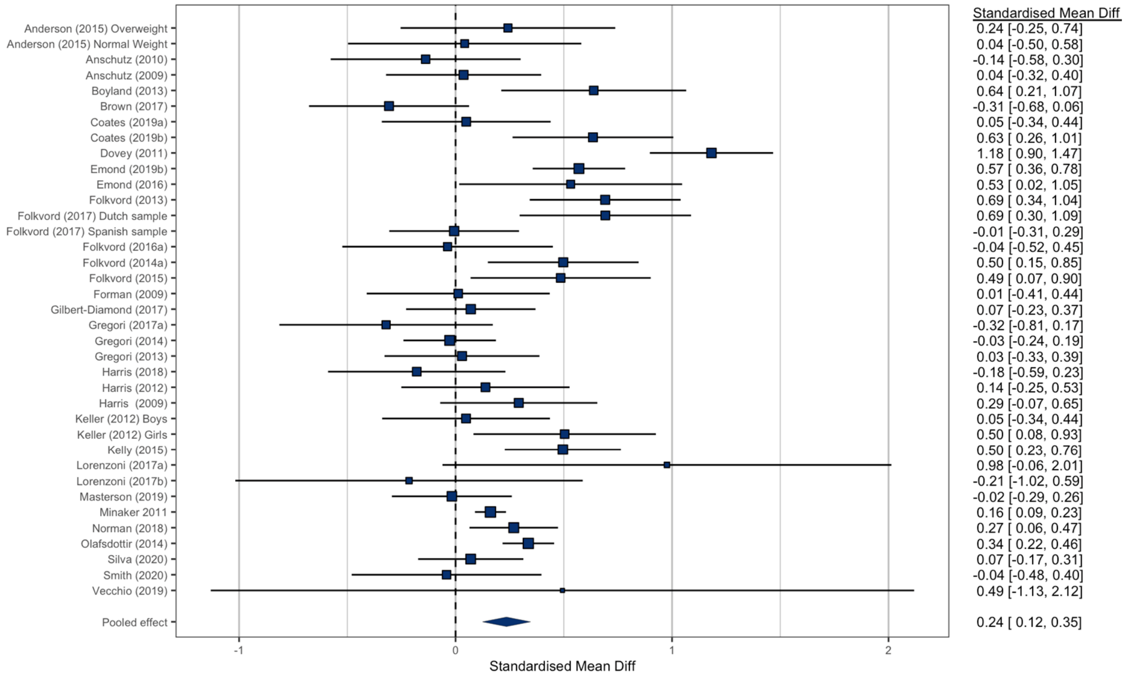

eFigure 4: Forest plot for pooled analyses of studies of more vs. less food marketing exposure with food intake outcomes

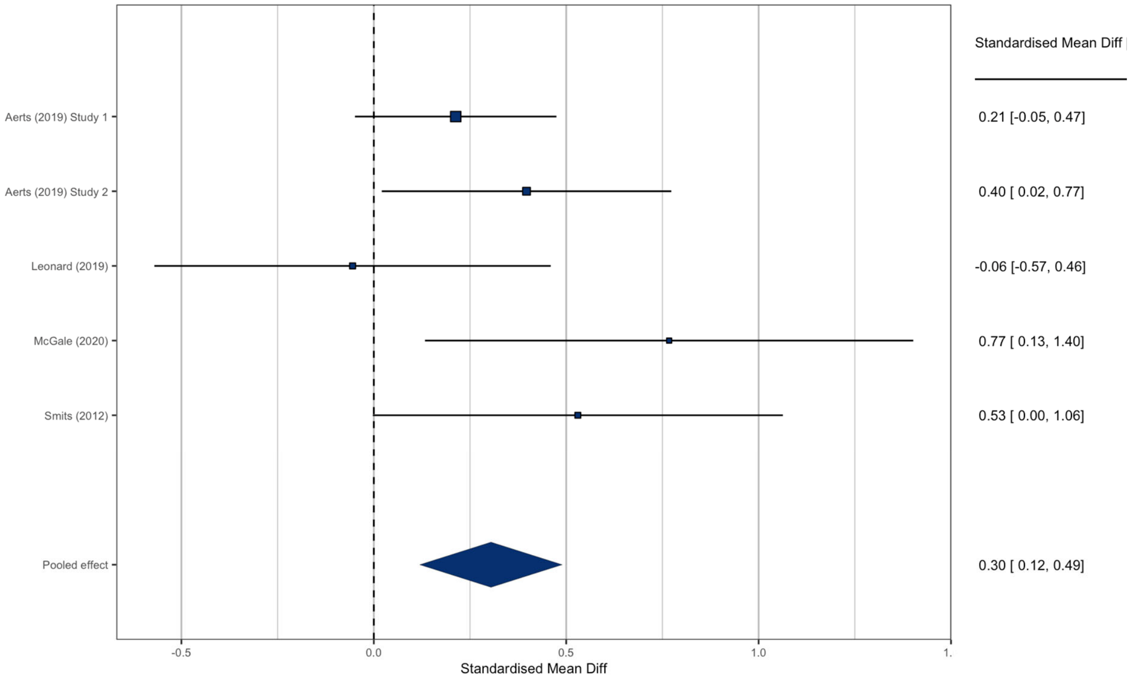

eFigure 5: Forest plot for pooled analyses of studies of more vs. less food marketing power with food intake outcomes

© 2022 Boyland et al. *JAMA Pediatrics*

Subgroup analyses by marketing channel

The effect size for television was SMD=0.21 [95% CI: .05 to .36: I^2=81.2%], the effect size for digital was SMD=0.32 [95% CI: .12 to .52: I^2=62.1%], and the effect size for packaging was SMD=0.20 [95% CI: .11 to .28: I^2=6.8%]. See eFigures 6, 7 and 8.

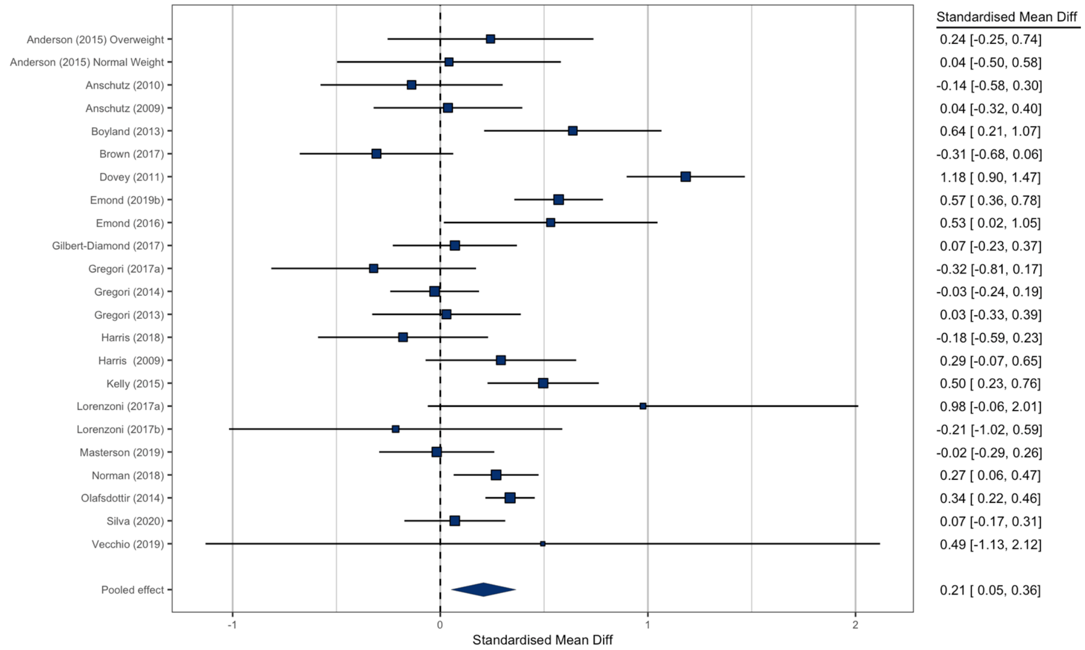

eFigure 6: Forest plot for pooled analyses of studies of television food marketing with food intake outcomes

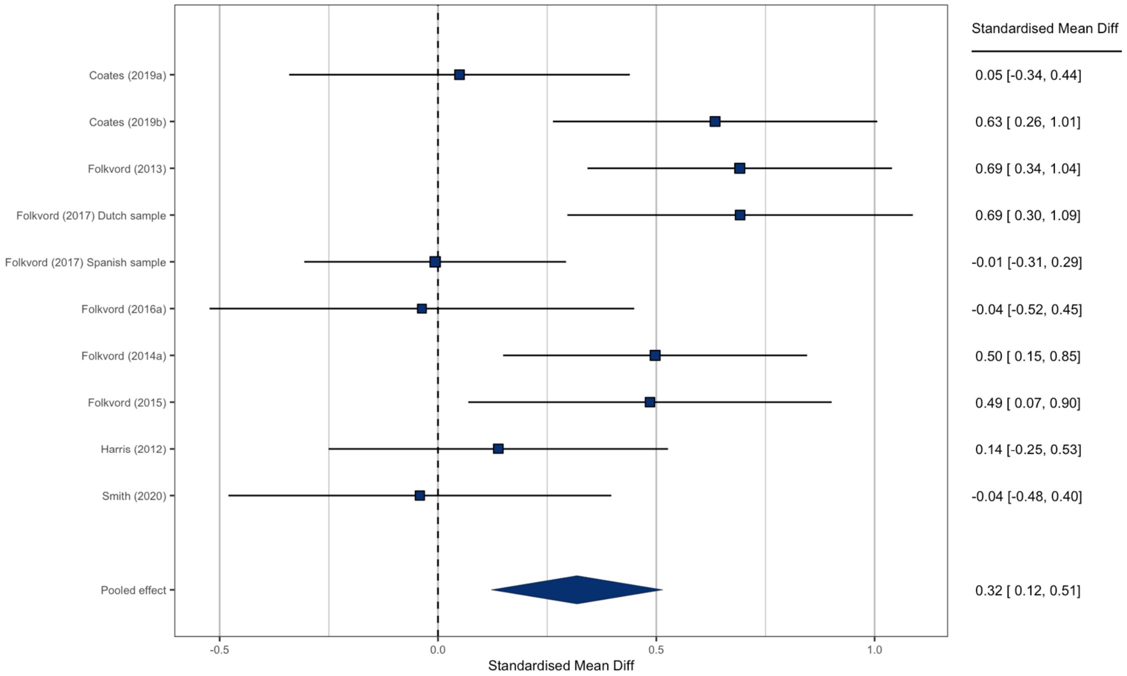

eFigure 7: Forest plot for pooled analyses of studies of digital food marketing with food intake outcomes

© 2022 Boyland et al. *JAMA Pediatrics*

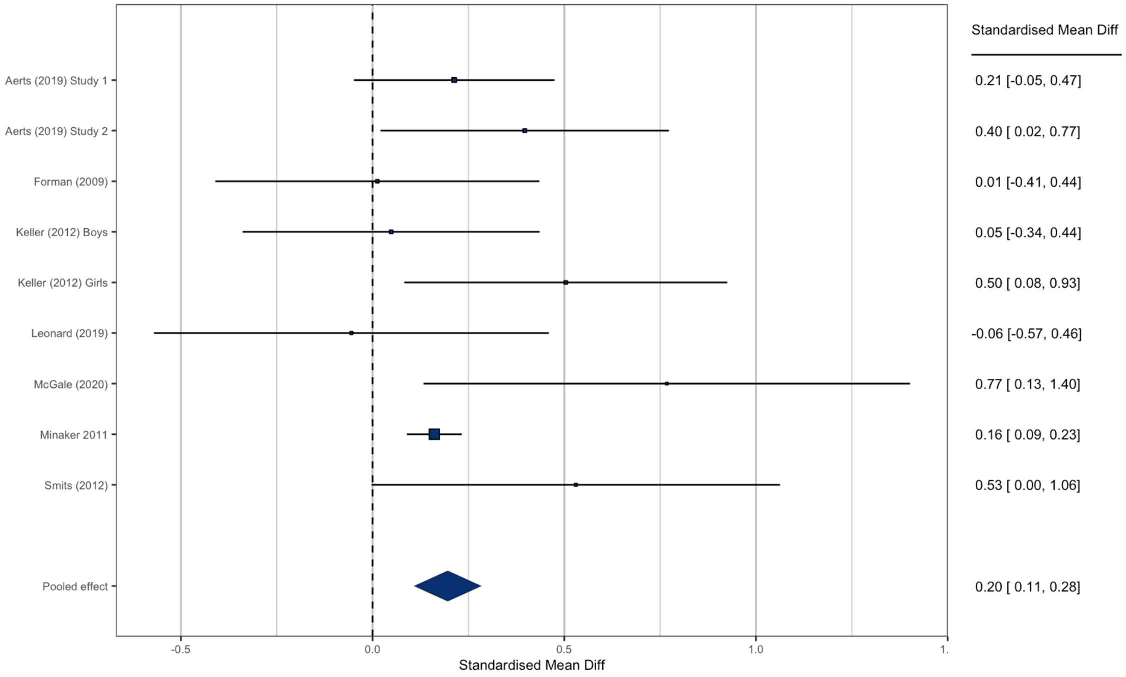

eFigure 8: Forest plot for pooled analyses of studies of packaging-based food marketing with food intake outcomes

P-curve analysis plot

The p-curve demonstrated clear right skew (see eFigure 9) which is demonstrative of evidential value (and lack of selective reporting).

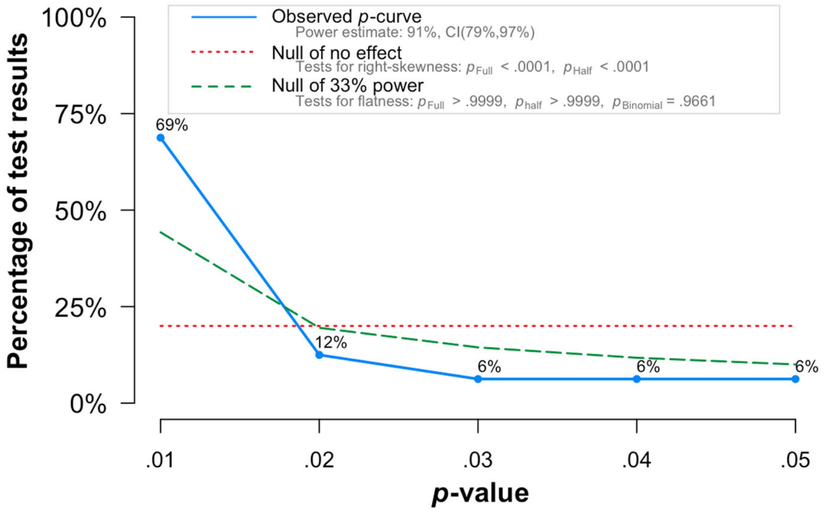

eFigure 9: Distribution of significant p-values from the p-curve test on food intake data.

© 2022 Boyland et al. *JAMA Pediatrics*

Food Choice

Sensitivity analyses

Leave-one-out analyses demonstrated little variability in the effect when individual studies were removed (OR 1.67-1.95), all models were statistically significant (ps<.001). Trim and fill analyses did not identify any hypothetical studies to impute, and no studies had a DFBETA value > 1. Egger's regression test was not significant (Z=1.54, p=.124).

GOSH analysis

The average I^2 across the 100,000 models was 74.06% and the average effect size was OR=1.702. A small number of pooled effect sizes were negative as can be seen from the histogram (eFigure 10). These were likely driven by the inclusion of one effect which was in the opposite direction from others.

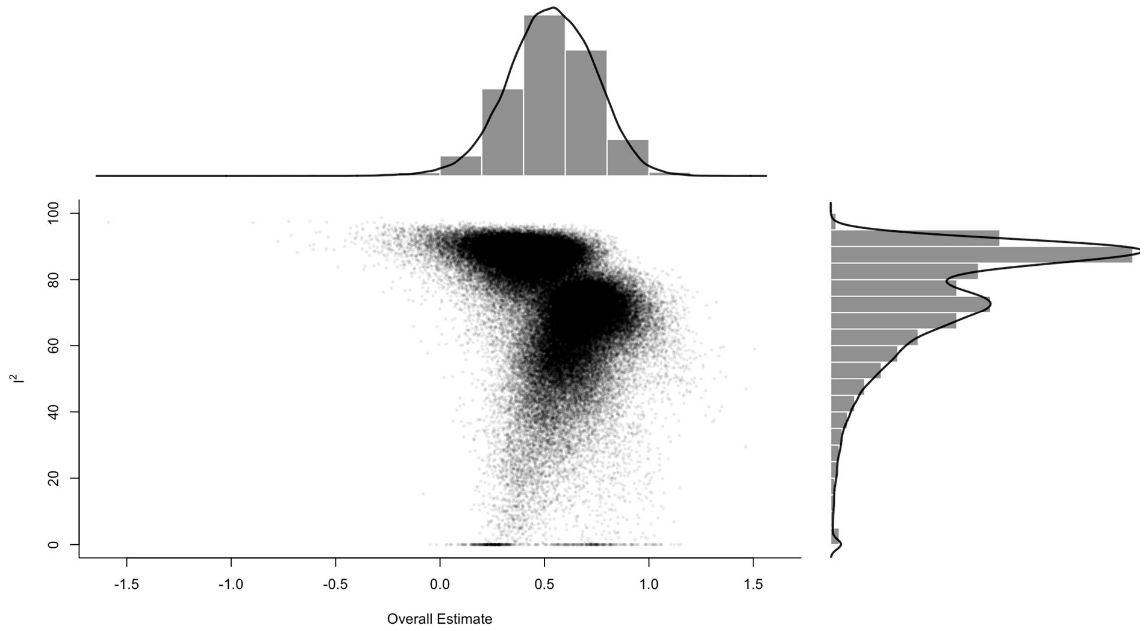

eFigure 10: GOSH plot for food choice meta-analysis

Subgroup analyses by risk of bias

There was no statistical evidence that study bias significantly moderated effect sizes in RCTs ($X^2(1)=2.07$, p=.15).

Subgroup analyses by study design

The effect size for RCTs (N=22; eFigure 11) was OR=1.97 [95% CI: 1.46 to 2.66: I^2=68.5%], the effect size for NRS (N=5; eFigure 12) was OR=0.56 [95% CI: 0.05 to 5.99: I^2=93.1%].

© 2022 Boyland et al. *JAMA Pediatrics*

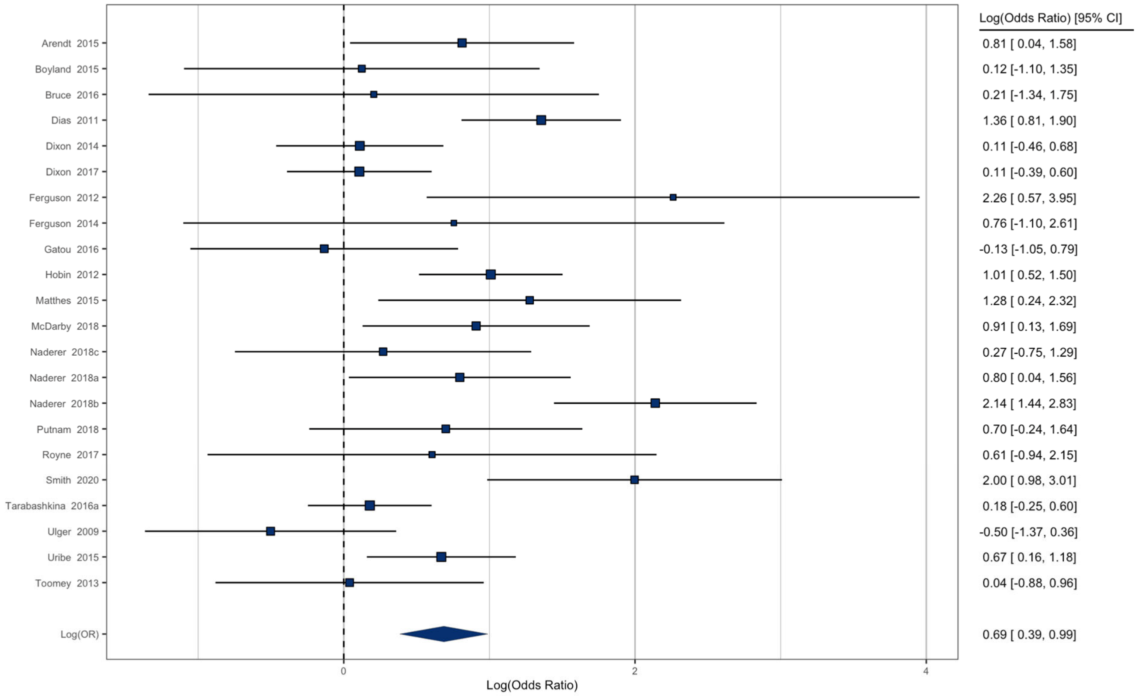

eFigure 11: Forest plot for pooled analyses of RCTs with food choice outcomes

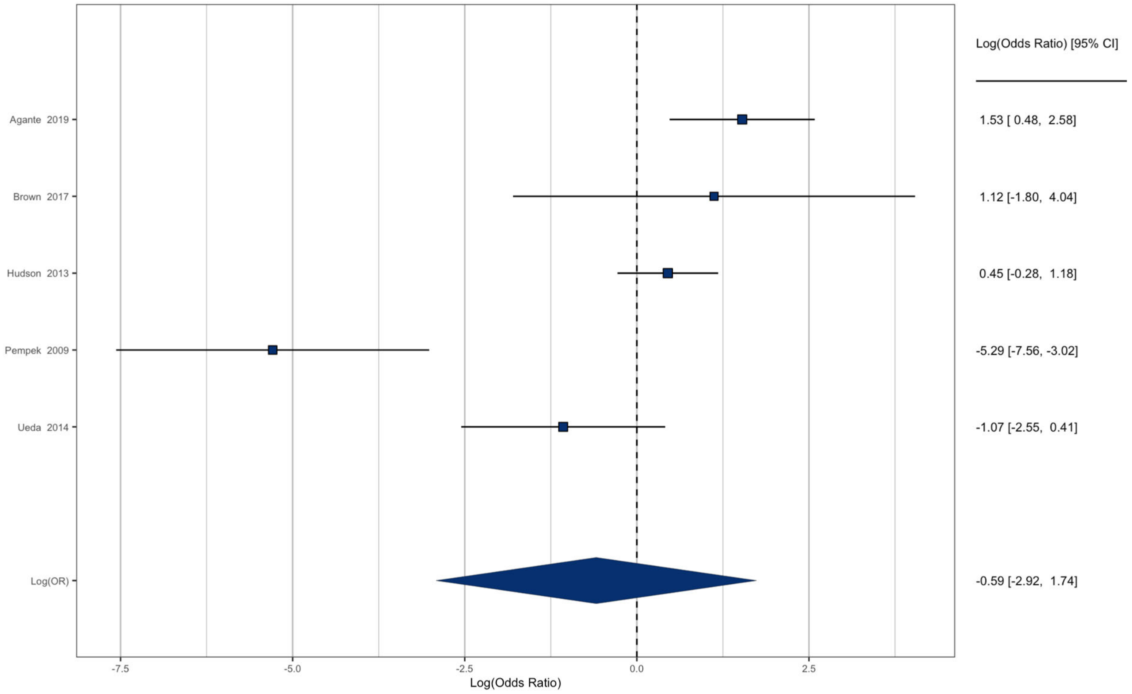

eFigure 12: Forest plot for pooled analyses of NRS with food choice outcomes

Subgroup analyses by marketing manipulation type

The effect size for exposure (N=23) was OR=1.75 [95% CI: 1.14 to 2.69: I^2 = 80.1%], the effect size for power was OR=1.67 [95% CI: 1.02 to 2.75: I^2=66.7%]. See eFigures 13 and 14.

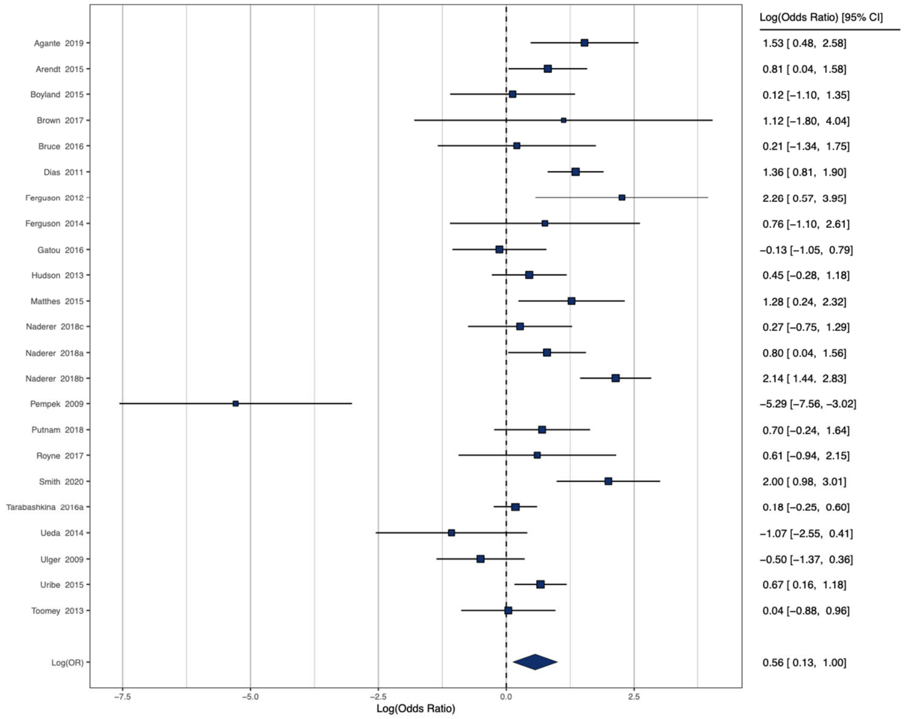

eFigure 13: Forest plot for pooled analyses of studies of more vs. less food marketing exposure with food choice outcomes

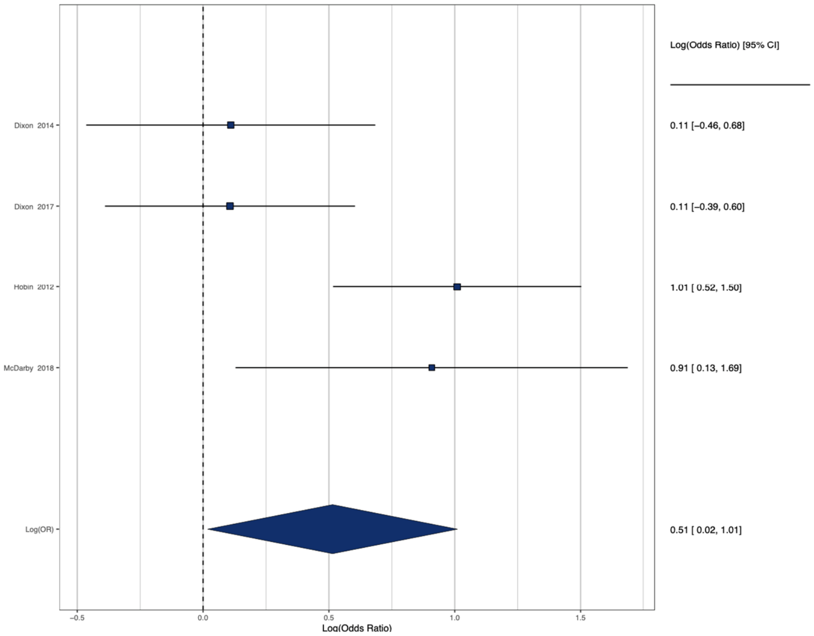

eFigure 14: Forest plot for pooled analyses of studies of more vs. less food marketing power with food choice outcomes

Subgroup analyses by marketing channel

The effect size for television (N=17) was OR=1.75 [95% CI: 1.17 to 2.61; I^2=61.8%], the effect size for digital (N=6) was OR=1.26 [95% CI: .18 to 8.58: I^2=97.2%], and the effect size for packaging (N=4) was OR=1.67 [95% CI: 1.02 to 2.75: I^2=66.7%]. See eFigures 15, 16 and 17.

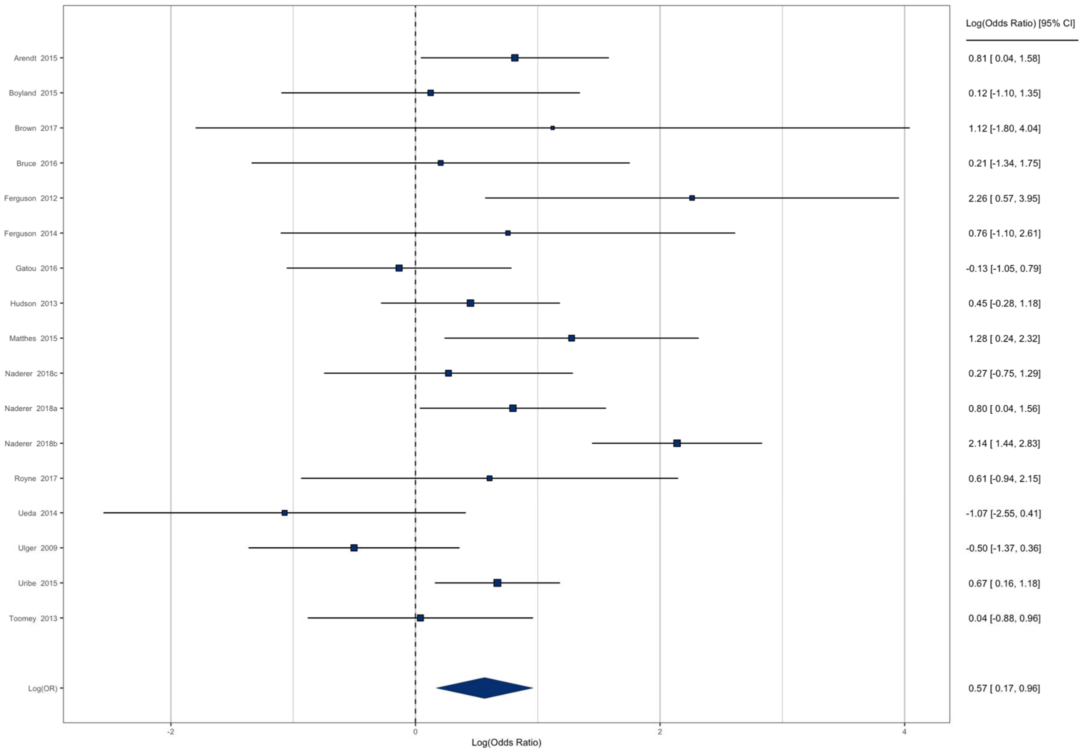

eFigure 15: Forest plot for pooled analyses of studies of television food marketing with food choice outcomes

© 2022 Boyland et al. *JAMA Pediatrics*

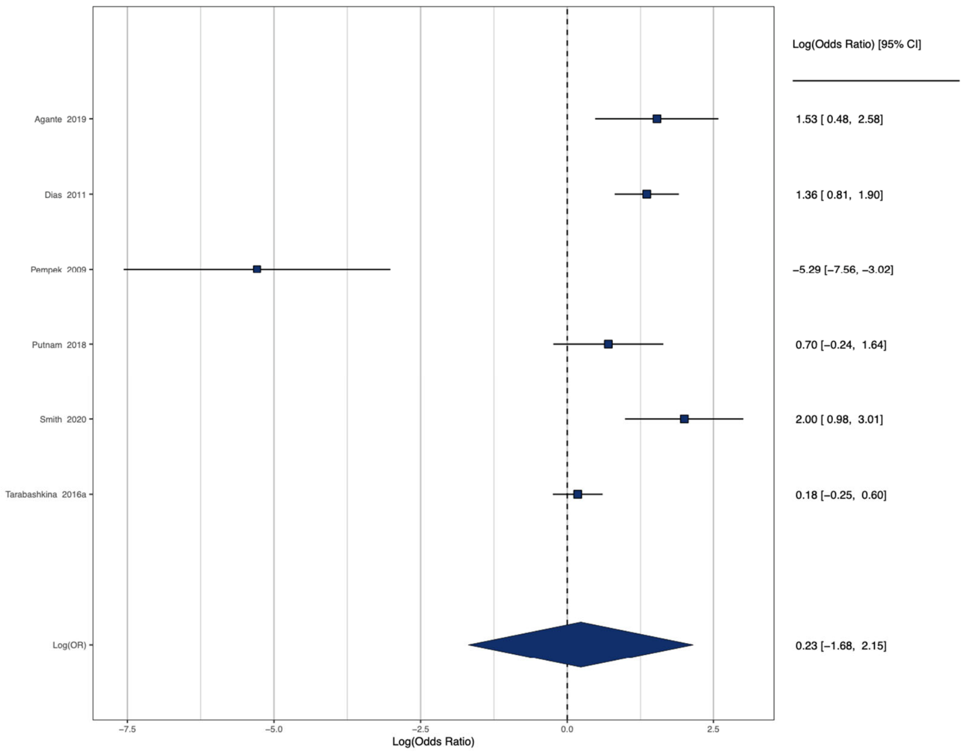

eFigure 16: Forest plot for pooled analyses of studies of digital food marketing with food choice outcomes

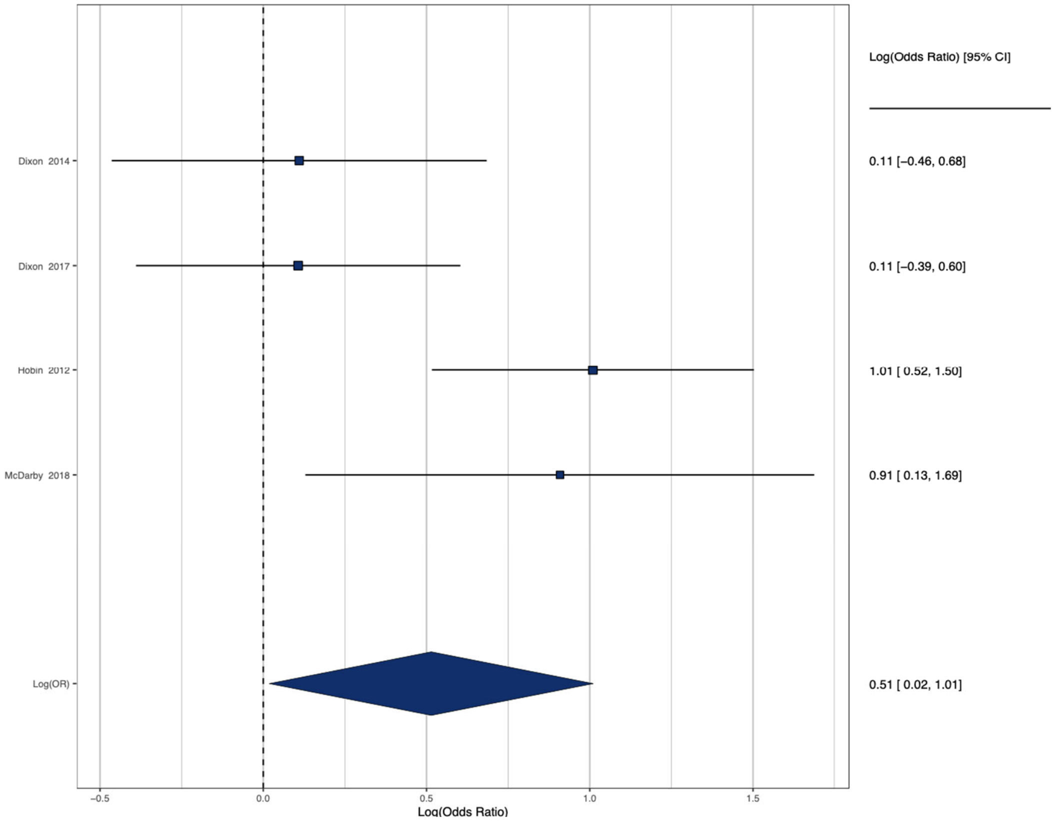

eFigure 17: Forest plot for pooled analyses of studies of packaging-based food marketing with food choice outcomes

P-curve analysis plot

The p-curve demonstrated clear right skew (see eFigure 18) which is demonstrative of evidential value (and lack of selective reporting).

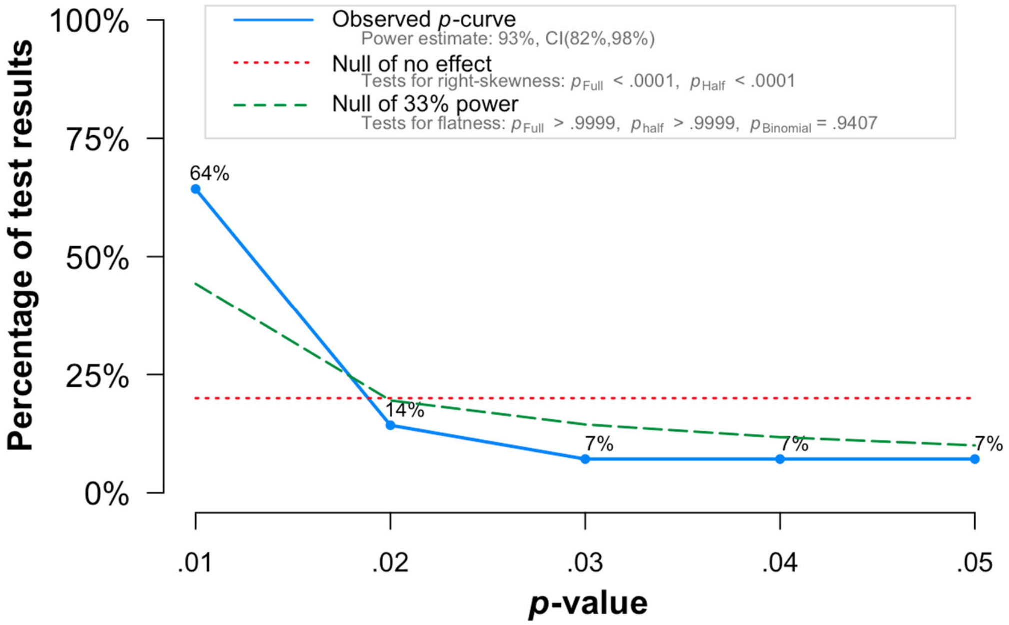

eFigure 18: Distribution of significant p-values from the p-curve test on food choice data.

© 2022 Boyland et al. *JAMA Pediatrics*

Food Preference

Sensitivity analyses

Leave-one-out analyses demonstrated variability in the effect when individual studies were removed (SMD=0.21-0.33), but all models were statistically significant (p-values p=.001 to p=.004). Trim and fill analyses did not identify any hypothetical studies to impute. Egger's regression test was significant (Z=3.14, p=.001). One study[14] had a DFBETA value >1. Removal of this study reduced the effect size and the heterogeneity (SMD=.21, 95% CI 0.11-0.31, I^2=65.3%]) but the overall effect remained significant (p<.001).

GOSH analysis

The average I^2 across the 100,000 models was 73.8% and the average effect size was SMD= 0.534. As can be seen from the histograms (eFigure 19), the majority of effect sizes were positive.

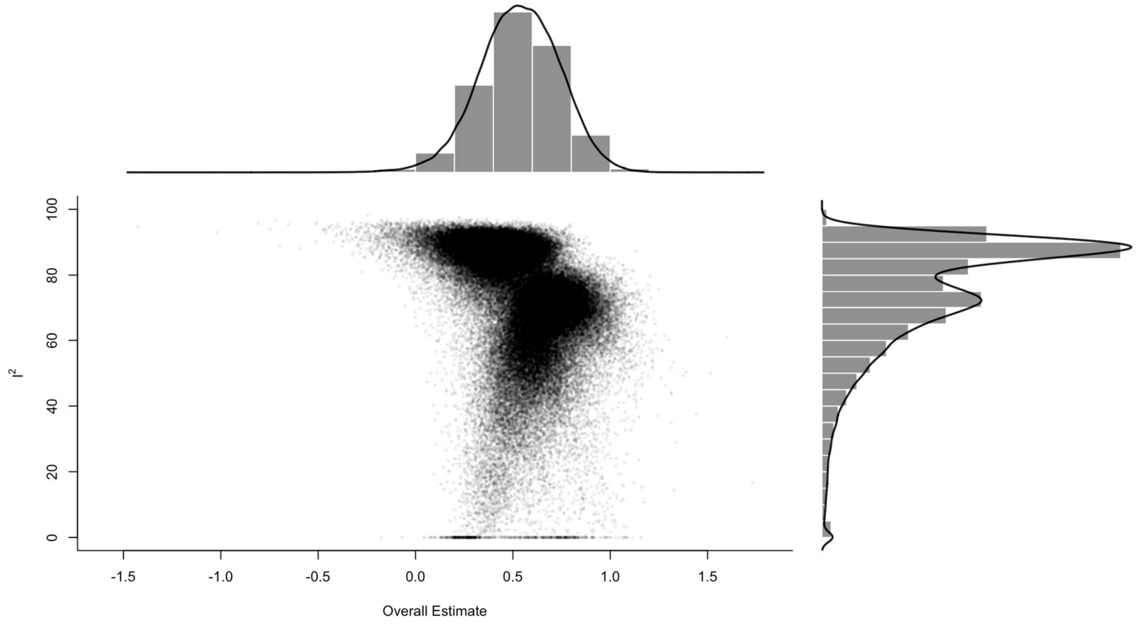

eFigure 19: GOSH plot for preference meta-analysis

Subgroup analyses by study design

The effect size for RCTs (N=9; eFigure 20) was SMD=0.38 [95% CI: .03 to .72: I^2=92.4%], the effect size for NRS (N=5; eFigure 21) was SMD=0.21 [95% CI: .07 to .36: I^2=74.1%].

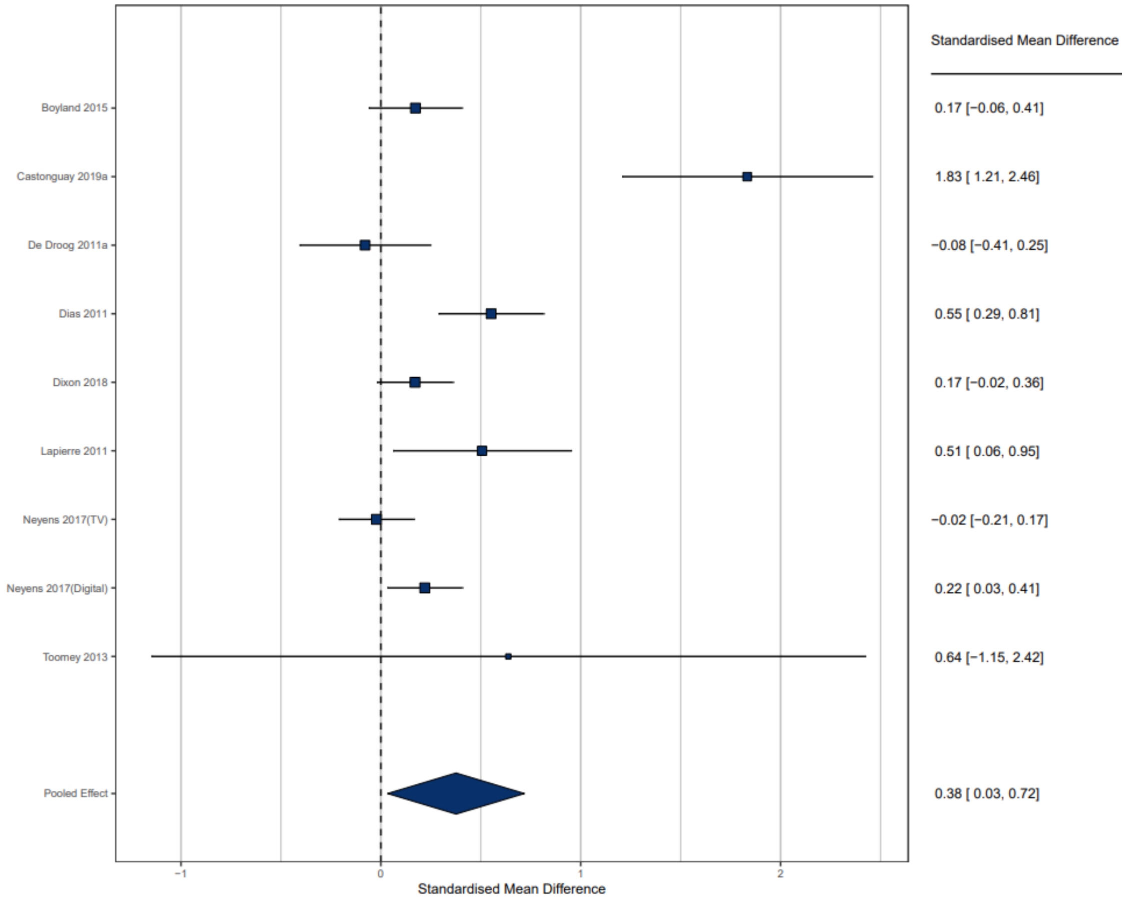

eFigure 20: Forest plot for pooled analyses of RCTs with food preference outcomes

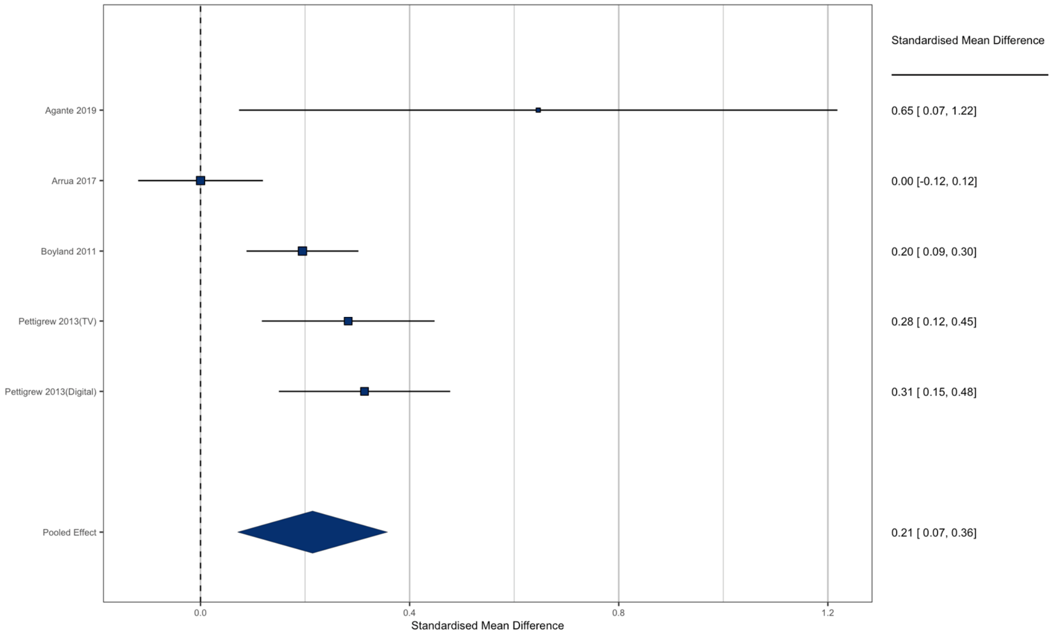

eFigure 21: Forest plot for pooled analyses of NRS with food choice outcomes

© 2022 Boyland et al. *JAMA Pediatrics*

Subgroup analyses by marketing manipulation type

The effect size for exposure was SMD=0.24 [95% CI: .14 to .33: I^2=50.2%], the effect size for power was SMD=0.53 [95% CI: -.30 to 1.36: I^2=96.2%]. See eFigures 22 and 23.

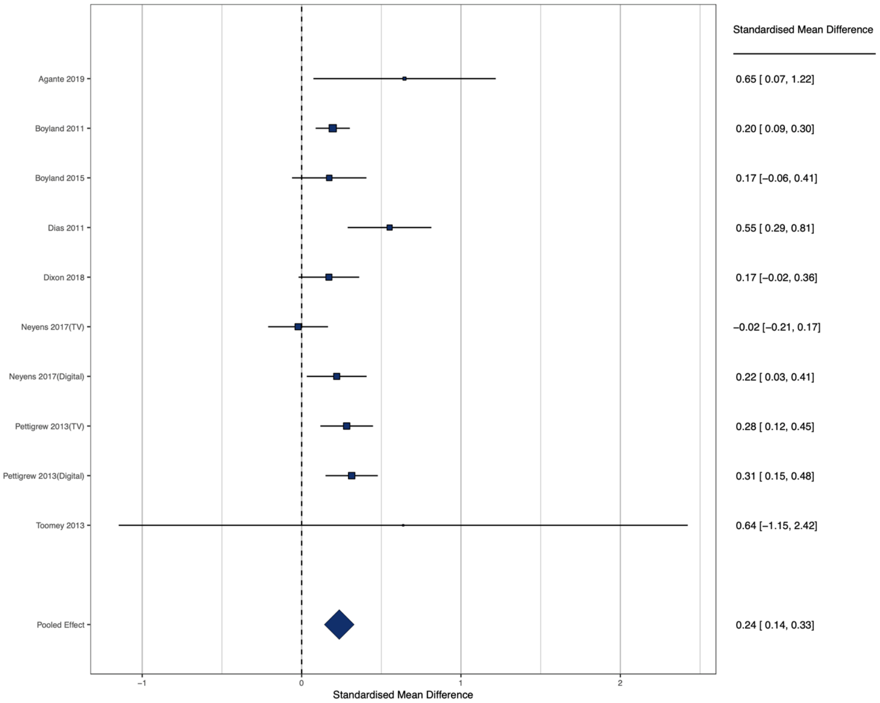

eFigure 22: Forest plot for pooled analyses of studies of more vs. less food marketing exposure with food preference outcomes

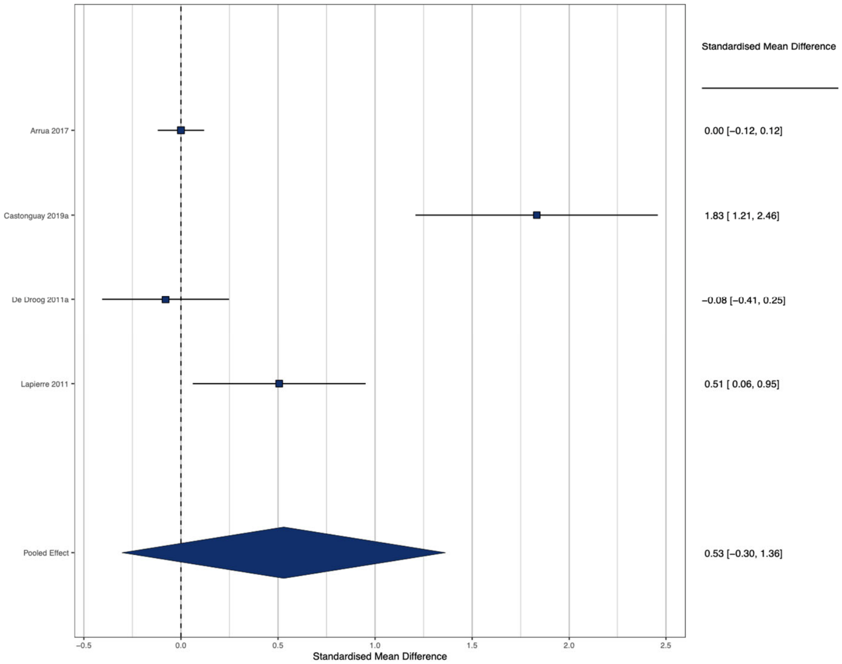

eFigure 23: Forest plot for pooled analyses of studies of more vs. less food marketing power with food preference outcomes

© 2022 Boyland et al. *JAMA Pediatrics*

Subgroup analyses by marketing channel

The effect size for television was SMD=0.44 [95% CI: -.15 to 1.03: I^2=97.9%], the effect size for digital was SMD=0.36 [95% CI: .20 to .53: I^2=45.4%], and the effect size for packaging was SMD=0.09 [95% CI: -.07 to .26: I^2=51.7%]. See eFigures 24, 25 and 26.

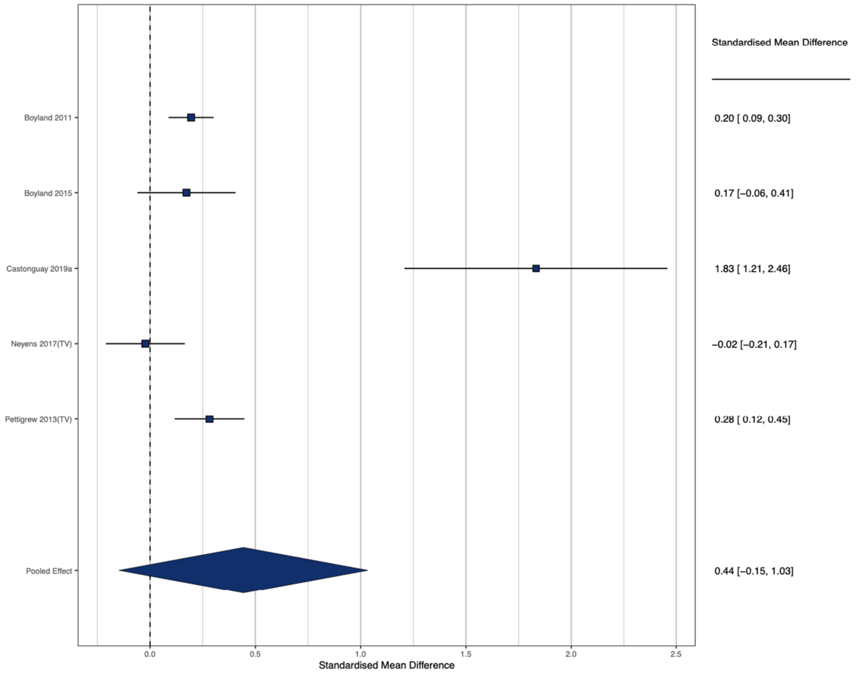

eFigure 24: Forest plot for pooled analyses of studies of television food marketing with food preference outcomes

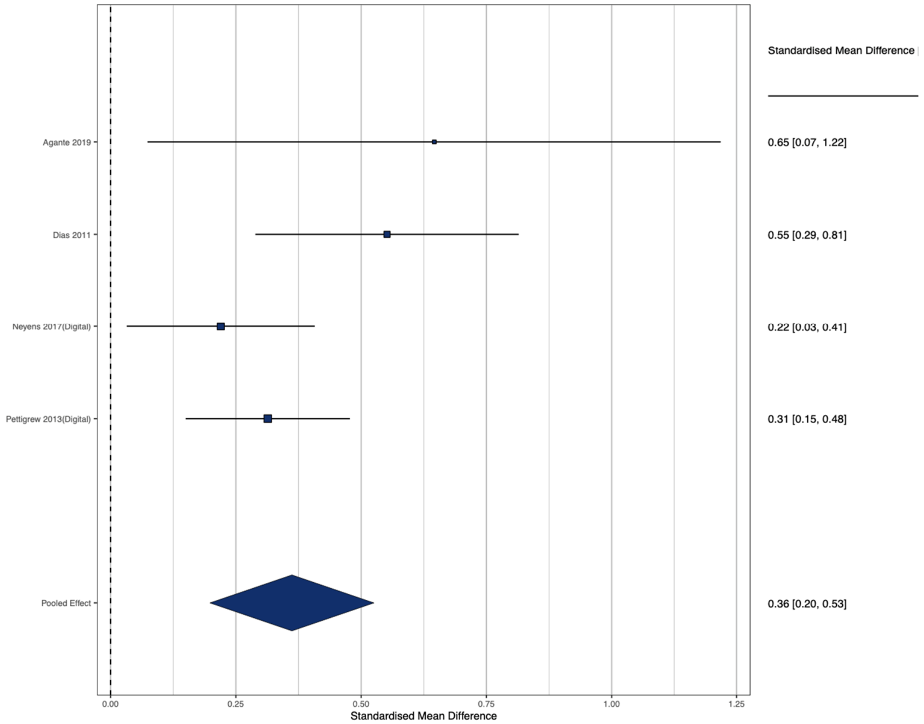

eFigure 25: Forest plot for pooled analyses of studies of digital food marketing with food preference outcomes

© 2022 Boyland et al. *JAMA Pediatrics*

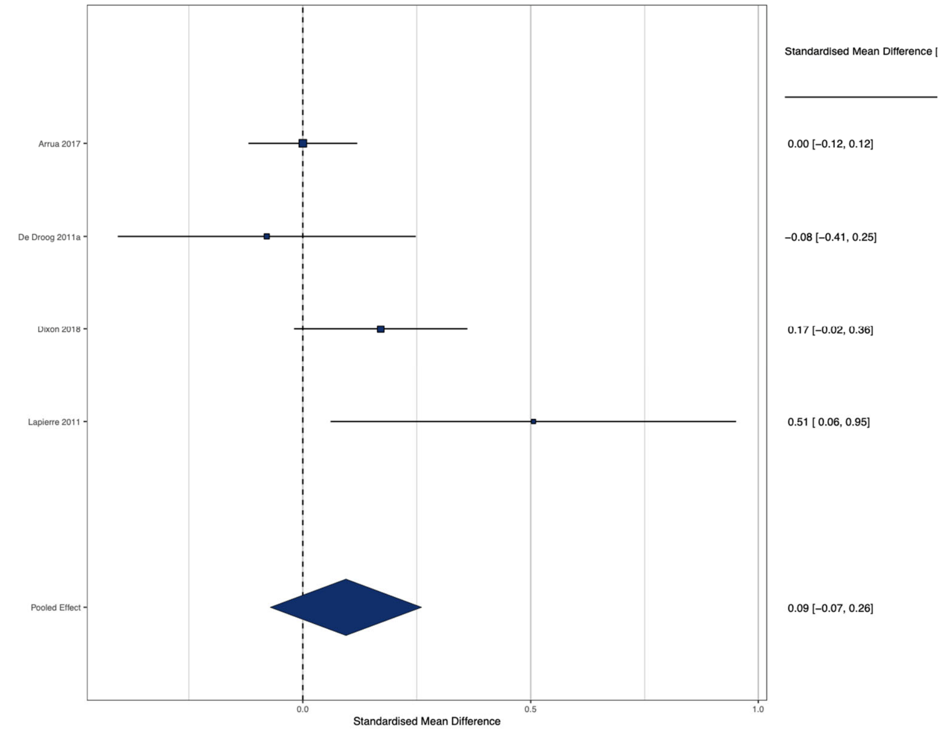

eFigure 26: Forest plot for pooled analyses of studies of packaging-based food marketing with food preference outcomes

P-curve analysis plot

The p-curve demonstrated clear right skew (see eFigure 27) which is demonstrative of evidential value (and lack of selective reporting).

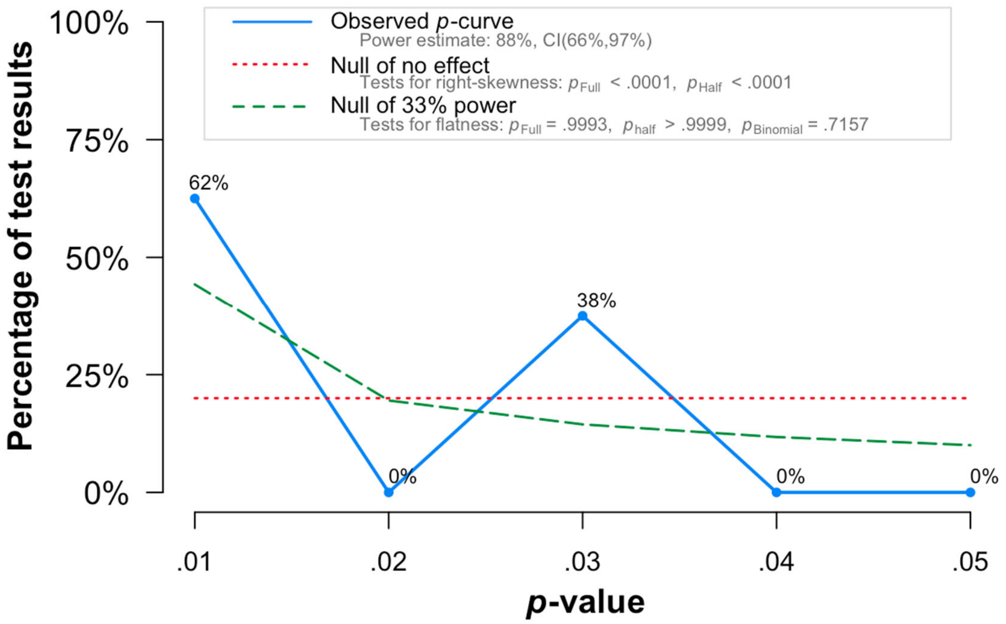

eFigure 27: Distribution of significant p-values from the p-curve test on food preference data.

Appendix 5: Data for outcomes not suitable for meta-analysis and harvest plot for vote counting by direction of effect outcomes

eTable 1: Data for purchasing outcome

Author	Year	Study design	Marketing format	Control exp.	Expt. Exp	Outcome	N# control	N# exp	Control value	Control SD	Exp. Value	Exp. SD	p value	Significance
Castetbon	2012	NRS	TV	No advertising for that brand	Child-targeted advertising for that brand	Number of buyers, median (≤ 1000)	202	17	869	25-75th percentiles (331-2236)	11165	25-75th percentiles (8684-19059)	NR	NR
Simoes	2014	NRS	Print	Control	Exposure to ad	Purchase intention rating (soft drinks, 5-point Likert)	136	133	3.54	1.253	3.86	1.106	NR	Non-sig
Drewnowski	2017	NRS	Other	No Happy Meal fruit dessert promotion	Happy Meal fruit dessert promotion	Percentage of orders for fruit dessert (2013)	NR	NR	19.4		20.1		<0.001	Sig higher in exp
Heard	2016	RCT	Packaging	No promotions	Unhealthy promotions	Unhealthy item purchased, M	21	15	1.81	95%CI 1.01-2.61)	2.4	95%CI 1.32-3.48	NR	Non-sig
Minaker	2011	NRS	Packaging	School has no snack logos	School has snack logos	Frequency of food and/or beverage purchasing	60%	40%			OR 1.61	95% CI 1.40-1.85	<0.0001	Sig higher in exp

eTable 2: Data for purchase request outcome

© 2022 Boyland et al. *JAMA Pediatrics*

Author	Year	Study design	Marketing format	Control exp.	Expt. Exp	Exp. Or Power	Outcome	N# control	N# exp	Control value	Control SD	Exp. Value	Exp. SD	Effect size	p value	Significance
Scully	2012	NRS	TV	No commercial TV viewing	Commercial TV >2h/d	Exposure	Ask for product advertised ≥ once in last month			1.00 (ref)		OR=1.61 (1.38, 1.88)			<0.001	
Dixon	2018	RCT	Packaging	Non-food branding	Unhealthy food branding	Exposure	Ask parents for product	289	273	3.49	1.49	3.39	1.5		>0.05	Non-sig
Dixon	2014	RCT	Packaging	No promotion	Sports celebrity endorsement	Power	Likelihood of asking to buy product	327	324	3.24	1.92	2.72	1.78		<0.01	Sig higher in control
Neyens	2017	RCT	Digital	No ad control	Advergame	Exposure	Pester intent	312	286	2.83	0.97	2.9	1	t(935) =0.91	0.363	Non-sig
Smits	2012	RCT	Packaging	Non-celebrity character	Celebrity character	Power	Asking for food item	NR	NR	3.63	2.95	6.53	2.66	F(1,53)=57.54	<0.0001	Sig higher in exp
De Droog	2011	RCT	Packaging	No character	Familiar character	Power	Purchase request intent	NR	NR	1.71	0.44	1.56	0.47		NR	NR

© 2022 Boyland et al. *JAMA Pediatrics*

eTable 3: Data for body weight outcome

Author	Year	Study design	Marketing format	Control exp.	Expt. Exp	Outcome	N# control	N# exp	Control value	Control SD	Exp. Value	Exp. SD	Effect size	p value	Significance
Minaker	2011	NRS	Packaging	School has no snack logos	School has snack logos	Children overweight or obese	60%	40%	NR	NR	NR	NR	OR 1.61 (95% CI 0.76-1.08)	0.274	Non-sig

© 2022 Boyland et al. *JAMA Pediatrics*

eTable 4: Data for dental caries outcome

Author	Year	Study design	Marketing format	Control exp.	Expt. Exp	Outcome	N# control	N# exp	Control value	Control SD	Exp. Value	Exp. SD	Effect size	p value	Significance
Silva	2020	NRS	TV	Don't watch TV commercials	Watch TV commercials	Caries experience (dmft + DMFT >1), n(%)	388	122	184 (47.4)	NR	64(52.5)	NR	OR brute 1.22 (0.81-1.84)	0.345	Non-sig
Ghimire	2013	NRS	TV	Don't watch TV commercials	Watch TV commercials	Caries experience (DMFT >1), n(%)	155	445	38(24.5)	NR	213(47.9)	NR		<0.001	Sig higher in exp

© 2022 Boyland et al. *JAMA Pediatrics*

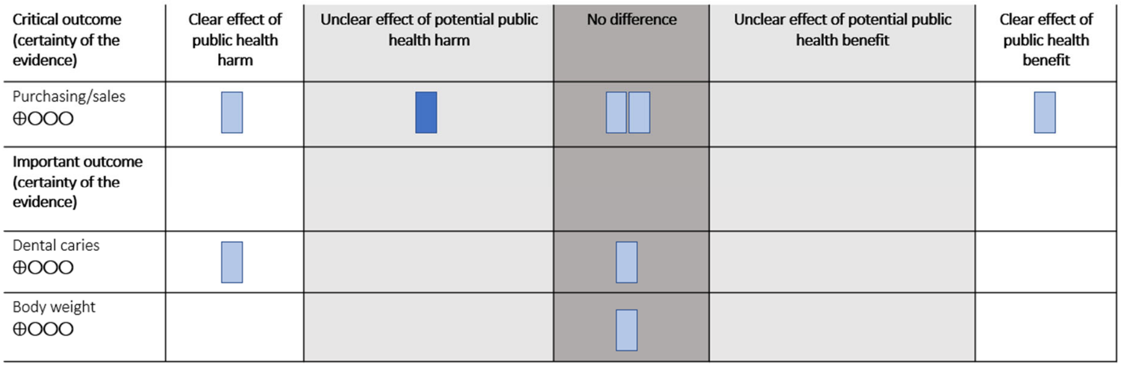

eFigure 28: **Harvest plot for outcomes analyses using vote counting by direction of effect**

Notes:

- Each bar represents <u>one</u> study
- Dark blue shading indicates a high quality study
- **Certainty of the evidence:** ⊕◯◯◯ very low, ⊕⊕◯◯ low, ⊕⊕⊕◯ moderate, ⊕⊕⊕⊕ high

© 2022 Boyland et al. *JAMA Pediatrics*

Appendix 6: GRADE evidence profiles

eTable 5: GRADE table for outcomes of pooled analyses only

© 2022 Boyland et al. *JAMA Pediatrics*

Certainty assessment							Number of subjects		Effect size (95% CI)	Certainty	Importance
No. of studies	Study design	Risk of bias	Inconsistency	Indirectness	Imprecision	Other considerations	Exp.	Control.			
Diet											
30	RCT	Not serious[1]	Serious[2]	Not serious	Not serious[3]	None	1456/2908 (50.1%)	1452/2908 (49.9%)	**SMD 0.20** (0.10 to 0.30)	⊕⊕⊕◯ MODERATE	Critical
11	Observational	Not serious[4]	Serious[5]	Not serious	Serious[6]	None	4245/8436 (50.3%)	4191/8436 (49.7%)	**SMD 0.34** (0.12 to 0.57)	⊕◯◯◯ VERY LOW	Critical
Choice											
22	RCT	Not serious[7]	Serious[8]	Not serious	Not serious[9]	None	1916/3838 (49.9%)	1922/3838 (50.1%)	**OR 1.97** (1.46 to 2.66)	⊕⊕⊕◯ MODERATE	Critical
5	Observational	Serious[10]	Serious[11]	Not serious	Very serious[12]	None	261/416 (62.7%)	155/416 (37.3%	**OR 0.56** (0.05-5.99)	⊕◯◯◯ VERY LOW	Critical
Preference											
8	RCT	Serious[13]	Serious[14]	Not serious	Serious[15]	None	894/1802 (49.6%)	908/1802 (50.4%)	**SMD 0.38** (0.03 to 0.72)	⊕◯◯◯ VERY LOW	Critical
4	Observational	Serious[16]	Serious[17]	Not serious	Serious[18]	None	1010/1972 (51.2%)	962/1972 (48.8%)	**SMD 0.21** (0.07 to 0.35)	⊕◯◯◯ VERY LOW	Critical

Explanations

© 2022 Boyland et al. *JAMA Pediatrics*

[1] Moderation analyses found no evidence that study bias significantly moderated the effect sizes of RCTs.
[2] High heterogeneity unexplained by sensitivity analyses.
[3] Effect size estimate as determined by the confidence interval parameters always reflecting public health harm from FNAB marketing and a small effect.
[4] Moderation analyses found no evidence that study bias significantly moderated the effect sizes of observational studies.
[5] High heterogeneity unexplained by sensitivity analyses.
[6] Effect size estimate as determined by the confidence interval parameters always reflecting public health harm from FNAB marketing but ranging from small to moderate.
[7] Moderation analyses found no evidence that study bias significantly moderated the effect sizes of RCTs.
[8] High heterogeneity unexplained by sensitivity analyses.
[9] Wide range of effect size estimate as determined by the confidence interval parameters but always reflecting public health harm from FNAB marketing.
[10] Low number of available studies and quality affected by potential for bias to be introduced by lack of sample representativeness, small sample size, lack of information on non-respondents, ascertainment of exposure and assessment of outcome.
[11] High heterogeneity unexplained by sensitivity analyses.
[12] Wide range of effect size estimate as determined by the confidence interval parameters, always reflecting public health harm from FNAB marketing but ranging from small to large size.
[13] Overall, no major concerns regarding risk of bias. Issues arise mostly from lack of disclosure of specific randomization processes and the potential for bias in the selection of results for reporting.
[14] High heterogeneity (although the effect did not change direction or significance). Sensitivity analyses demonstrated that there was variability in the effect when individual studies were removed but these analyses do not provide a public health relevant explanation for the heterogeneity so rating down for heterogeneity is still appropriate.
[15] Effect size estimate range >0.50 as determined by the confidence interval parameters, always reflecting public health harm from FNAB marketing but ranging from small to moderate size.
[16] Low number of available studies and quality affected by potential for bias to be introduced by lack of sample representativeness, small sample size, lack of information on non-respondents, and assessment of outcome.
[17] High heterogeneity (although the effect did not change direction or significance). Sensitivity analyses demonstrated that there was variability in the effect when individual studies were removed but these analyses do not provide a public health relevant explanation for the heterogeneity so rating down for heterogeneity is still appropriate.
[18] Effect size estimate as determined by the confidence interval parameters always reflecting public health harm from FNAB marketing but ranging from small to moderate.

© 2022 Boyland et al. *JAMA Pediatrics*

eTable 6: GRADE table for outcomes including all data

© 2022 Boyland et al. *JAMA Pediatrics*

Certainty assessment							Impact	Certainty	Importance
**No. of studies**	**Study design**	**Risk of bias**	**Inconsistency**	**Indirectness**	**Imprecision**	**Other considerations**			
**DIET**									
31	RCT	Not serious[1]	Serious[2]	Not serious[3]	Not serious	None	**Overall, the RCT evidence shows that FNAB marketing likely increases dietary intake slightly**. Level of certainty is affected by the serious inconsistency of the data, due to heterogeneity of study design (stimuli, populations, units of measurement, etc).	⨁⨁⨁◯ MODERATE	Critical
15	Observational	Not serious[4]	Serious[5]	Not serious[6]	Serious[7]	None	**Overall, the observational evidence is very uncertain about the effect of FNAB marketing on dietary intake**. Level of certainty is affected by the serious inconsistency of the data (as above) and the lack of precision in relation to the effect size.	⨁◯◯◯ VERY LOW	Critical
**CHOICE**									
27	RCT	Not serious[8]	Serious[9]	Not serious[10]	Not serious[11]	None	**Overall, the RCT evidence shows that FNAB marketing likely increases choice of foods and non-alcoholic beverages**. Level of certainty is downgraded by the serious inconsistency of the data, due to heterogeneity of study design (stimuli, populations, units of measurement, etc).	⨁⨁⨁◯ MODERATE	Critical
10	Observational	Serious[12]	Serious[13]	Not serious[14]	Very serious[15]	None	**Overall, the observational evidence is very uncertain about the effect of FNAB marketing on choice of foods and non-alcoholic beverages.** Level of certainty is affected by potential for bias, the serious inconsistency of the data (as above) and the substantial lack of precision in relation to the effect size.	⨁◯◯◯ VERY LOW	Critical
**PREFERENCE**									
12	RCT	Serious[16]	Serious[17]	Not serious[18]	Serious[19]	None	**Overall, the RCT evidence is very uncertain about the effect of FNAB marketing on preference for foods and non-alcoholic beverages.** Level of certainty is affected by potential for bias,	⨁◯◯◯ VERY LOW	Critical

							the serious inconsistency of the data, due to heterogeneity of study design (stimuli, populations, units of measurement, etc) and the imprecision of the effect size estimate in pooled analyses.		
8	Observational	Serious[20]	Serious[21]	Not serious[22]	Serious[23]	None	**Overall, the observational evidence is very uncertain about the effect of FNAB marketing on preference for foods and non-alcoholic beverages**. Level of certainty is affected by the potential for bias, the serious inconsistency of the data and the imprecision of the effect size estimate in pooled analyses.	⊕◯◯◯ VERY LOW	Critical
**PURCHASE**									
1	RCT	Serious[24]	Not serious	Not serious	Very serious[25]	None	**Overall, the RCT evidence is very uncertain about the effect of FNAB marketing on purchase of foods and non-alcoholic beverages**. This assessment is based on a single RCT with some concerns of bias, the level of certainty is affected by this bias and imprecision of the non-significant effect (small sample and wide confidence intervals). No pooled analysis is available for this outcome.	⊕◯◯◯ VERY LOW	Critical
4	Observational	Serious[26]	Not serious	Not serious[27]	Not serious	None	**Overall, the observational evidence is very uncertain about the effect of FNAB marketing on purchase of foods and non-alcoholic beverages.** The level of certainty is affected by the risk of bias due to issues with study quality. No pooled analysis is available for this outcome.	⊕◯◯◯ VERY LOW	Critical
**PURCHASE REQUESTS**									
5	RCT	Serious[28]	Not serious[29]	Not serious	Not serious	None	**Overall, the RCT evidence shows that FNAB marketing likely increases food and non-alcoholic beverage purchase requests**. The level of certainty is affected by serious risk of bias.	⊕⊕⊕◯ MODERATE	Important
1	Observational	Serious[30]	Not serious	Not serious	Not serious	None	**Overall, the observational evidence is very uncertain about the effect of FNAB marketing on purchase requests for**	⊕◯◯◯ VERY LOW	Important

© 2022 Boyland et al. *JAMA Pediatrics*

							**foods and non-alcoholic beverages**. This assessment is based on a single observational study of moderate quality. The sample size was large (>12,000 participants) and effects consistent (OR 1.5-3.2) across three marketing formats but below the typical threshold for a 'large effect', data speak directly to the research question but in the absence of further studies to corroborate this effect certainty remains very low.		
**BODY WEIGHT**									
0	RCT	-	-	-	-	-	-	-	-
1	Observatio nal	Serious[31]	Not serious	Serious[32]	Not serious	None	**Overall, the observational evidence is very uncertain about the effect of FNAB marketing on body weight outcomes**. This outcome is assessed based on a single cross-sectional study with a large sample size (>4,500). No effect was found, and the study had a serious risk of bias and serious indirectness. Therefore, overall, the level of certainty in this outcome is very low.	⊕◯◯◯ VERY LOW	Important
**DENTAL CARIES**									
0	RCT	-	-	-	-	-	-	-	-
2	Observatio nal	Serious[33]	Serious[34]	Serious[35]	Not serious	None	**Overall, the observational evidence is very uncertain about the effect of FNAB marketing on dental health outcomes**. This outcome is assessed from just two studies in which one found a significant effect across two measures and the other found no effect. Level of certainty is affected by serious risk of bias, inconsistency, and indirectness of the intervention in providing direct evidence for the research question.	⊕◯◯◯ VERY LOW	Important

Explanations

[1] Moderation analyses found no evidence that study bias significantly moderated the effect sizes of RCTs, the non-pooled RCT also had "some concerns" (like the majority) so would be unlikely to affect this overall outcome.

© 2022 Boyland et al. *JAMA Pediatrics*

[2] High heterogeneity in pooled data unexplained by sensitivity analyses, significant effect of exposure found in non-pooled RCT consistent with pooled effect but not sufficient to ameliorate concerns.
[3] No serious indirectness but note variability in the intervention and outcome measures.
[4] Moderation analyses found no evidence that study bias significantly moderated the effect sizes of observational studies, non-pooled studies all moderate quality so unlikely to affect this overall outcome.
[5] High heterogeneity in pooled data unexplained by sensitivity analyses, effect found in two of four non-pooled studies.
[6] No serious indirectness but note variability in the intervention and outcome measures.
[7] Effect size estimates for pooled studies as determined by the confidence interval parameters always reflecting public health harm from FNAB marketing but ranging from small to moderate effect size. Non-pooled studies show some variability in effect size (RR 1.19 and OR 1.31) and two studies showed no effect so unlikely to substantially refine the point estimate.
[8] Moderation analyses found no evidence that study bias significantly moderated the effect sizes of RCTs, the non-pooled RCTs also mostly had "some concerns" (one small study was high risk) so would be unlikely to affect this overall outcome.
[9] High heterogeneity in pooled data unexplained by sensitivity analyses, effect of same direction to pooled analysis found in three of four non-pooled studies but in two studies another comparison was non-significant, or an effect was found in the opposite direction.
[10] No serious indirectness but note variability in the intervention and outcome measures.
[11] Wide range of effect size estimate as determined by the confidence interval parameters of pooled analysis (albeit always reflecting public health harm from FNAB marketing and four of five RCTs not in the pooled analysis found a significant effect in the same direction).
[12] Six of ten studies of low-moderate quality (NOS $\leq$ 5), issues arising from sample representativeness, lack of justification of sample size, lack of information on non-respondents, use of recall to ascertain exposure, lack of control of confounding factors and the lack of information over the blinding of assessment.
[13] High heterogeneity in pooled data unexplained by sensitivity analyses, effect found in three of five non-pooled studies.
[14] No serious indirectness but note variability in the intervention and outcome measures.
[15] Very wide confidence intervals around effect estimate in pooled analyses (0.05-5.99), non-pooled studies show variation in effect (including two reporting no effect) so would be unlikely to substantially refine the point estimate.
[16] Some concerns of bias, issues arising from lack of disclosure of specific randomization processes and potential deviations from intended interventions (note that p curve analysis supports lack of selective reporting of results).
[17] High heterogeneity in pooled data unexplained by sensitivity analyses, effect found in one of three non-pooled studies.
[18] No serious indirectness but note variability in the intervention and outcome measures.
[19] Effect size estimate range >0.50 as determined by the confidence interval parameters in pooled analysis, although always reflecting public health harm from FNAB marketing.
[20] Study quality issues arising from concerns over sample representativeness, lack of justification of sample size, and lack of information on non-respondents or the blinding of assessment.
[21] High heterogeneity in pooled data unexplained by sensitivity analyses, effect found in two of four non-pooled studies.
[22] No serious indirectness but note variability in the intervention and outcome measures.
[23] Effect size estimates as determined by the confidence interval parameters in pooled analysis always reflecting public health harm from FNAB marketing but ranging from small to moderate size. Non-pooled studies show variation in effect (including two reporting no effect) so would be unlikely to substantially refine the point estimate.
[24] Some concerns of bias, issues arising from lack of disclosure of specific randomization processes and potential deviations from intended interventions.
[25] Small sample size (<100) and wide confidence intervals.
[26] Study quality was affected mostly by lack of justification for sample size and lack of information on non-respondents, but some studies also failed to control for potential confounding factors or used non-validated measures of outcome assessment.
[27] No serious indirectness but note variability in the intervention and outcome measures.
[28] Most studies had "some concerns" of bias (also one high risk and one low risk), issues arise mostly from lack of disclosure of specific randomization processes as well as some potential deviations from intended interventions and the potential for bias in the selection of results for reporting.

© 2022 Boyland et al. *JAMA Pediatrics*

[29] The combination of p-values was statistically significant (ps < .001) in all model iterations.
[30] Study deemed to be of moderate quality, issues arising from lack of justification of sample size, lack of information on non-respondents, and use of recall to ascertain exposure.
[31] Study deemed to be of moderate quality, issues arising from lack of justification of sample size, lack of information on non-respondents, and use of recall to ascertain exposure.
[32] The measurement of exposure to marketing reflects awareness of the presence of snack and/or beverage logos within the school environment rather than an individual level of exposure to that marketing.
[33] One study was of high quality, but the other was low quality due to issues arising from lack of justification of sample size, lack of information on non-respondents, use of recall to ascertain exposure, lack of control of confounding factors and the lack of information over the blinding of assessment.
[34] The direction and magnitude of effect varied across the two studies. The results showed either no change or a significant impact of food marketing on dental caries experience.
[35] Ascertainment of exposure reflected attention paid to commercials rather than actual exposure, so the evidence was judged to have serious indirectness despite the consistency of outcome measurement across studies.

© 2022 Boyland et al. *JAMA Pediatrics*

References for supplementary material

1. Murad MH, Mustafa RA, Schünemann HJ, Sultan S, Santesso N. Rating the certainty in evidence in the absence of a single estimate of effect. *Evidence Based Medicine* 2017; **22**(3): 85-7.
2. McKenzie JE, Brennan SE. Chapter 12: Synthesizing and presenting findings using other methods. In: Higgins JPT, Thomas J, Chandler J, et al., eds. Cochrane Handbook for Systematic Reviews of Interventions version 62 (updated February 2021) Available from wwwtrainingcochraneorg/handbook: Cochrane; 2021.
3. Ogilvie D, Egan M, Hamilton V, Petticrew M. Promoting walking and cycling as an alternative to using cars: systematic review. *BMJ* 2004; **329**(7469): 763.
4. Burns J, Polus S, Brereton L, et al. Looking beyond the forest: Using harvest plots, gap analysis, and expert consultations to assess effectiveness, engage stakeholders, and inform policy. *Research Synthesis Methods* 2018; **9**(1): 132-40.
5. Cohen J. Statistical power analysis for the behavioural sciences. 2nd edition ed. Hillsdale (HJ): Erlbaum; 1988.
6. Higgins JPT, Thomas J, Chandler J, et al. Cochrane Handbook for Systematic Reviews of Interventions version 6.2 (updated February 2021). Available from www.training.cochrane.org/handbook.; 2021.
7. Balk EM, Earley A, Patel K, Trikalinos TA, Dahabreh IJ. Empirical Assessment of Within-Arm Correlation Imputation in Trials of Continuous Outcomes. Methods Research Report. (Prepared by the Tufts Evidence-based Practice Center under Contract No. 290-2007-10055-I.) AHRQ Publication No. 12(13)-EHC141-EF. Rockville, MD: Agency for Healthcare Research and Quality. 2012.
8. Chinn S. A simple method for converting an odds ratio to effect size for use in meta-analysis. *Stat Med* 2000; **19**(22): 3127-31.
9. Deeks JJ, Higgins JPT, Altman DG. Chapter 10: Analysing data and undertaking meta-analyses. In: Higgins JPT, Thomas J, Chandler J, et al., eds. Cochrane Handbook for Systematic Reviews of Interventions version 62 (updated February 2021): Cochrane; 2021.
10. Nolan SJ, Hambleton I, Dwan K. The Use and Reporting of the Cross-Over Study Design in Clinical Trials and Systematic Reviews: A Systematic Assessment. *PLoS One* 2016; **11**(7): e0159014.
11. Elbourne DR, Altman DG, Higgins JP, Curtin F, Worthington HV, Vail A. Meta-analyses involving cross-over trials: methodological issues. *Int J Epidemiol* 2002; **31**(1): 140-9.
12. Simonsohn U, Nelson LD, Simmons JP. P-curve: A key to the file drawer. *Journal of Experimental Psychology: General* 2013; **Forthcoming. Available at SSRN: https://ssrn.com/abstract=2256237**.
13. Olkin I, Dahabreh IJ, Trikalinos TA. GOSH - a graphical display of study heterogeneity. *Res Synth Methods* 2012; **3**(3): 214-23.
14. Castonguay J. Sugar and Sports: Age Differences in Children's Responses to a High Sugar Cereal Advertisement Portraying Physical Activities. *Communication Research* 2019a; **46**(5): 579-96.
